# Prioritization of potential vaccine targets using comparative proteomics and designing of the chimeric multi-epitope vaccine against *Pseudomonas aeruginosa*

**DOI:** 10.1038/s41598-019-41496-4

**Published:** 2019-03-27

**Authors:** Vandana Solanki, Monalisa Tiwari, Vishvanath Tiwari

**Affiliations:** Department of Biochemistry, Central University of Rajasthan, Ajmer, 305817 India

## Abstract

Multidrug-resistant *Pseudomonas aeruginosa* is one of the worldwide health problems involved in elevated mortality and morbidity. Therefore, it is important to find a therapeutic for this pathogen. In the present study, we have designed a chimeric vaccine against *P. aeruginosa* with the help of comparative proteomics and reverse vaccinology approaches. Using comparative subtractive proteomic analysis of 1,191 proteomes of *P. aeruginosa*, a total of twenty unique non-redundant proteomes were selected. In these proteomes, fifteen outer membrane proteins (OMPs) of *P. aeruginosa* were selected based on the basis of hydrophilicity, non-secretory nature, low transmembrane helix (<1), essentiality, virulence, pathway association, antigenic, and protein-protein network analysis. Reverse vaccinology approach was used to identify antigenic and immunogenic MHC class I, MHC class II and B cell epitopes present in the selected OMPs that can enhance T cell and B cell mediated immunogenicity. The selected epitopes were shortlisted based on their allergenicity, toxicity potentials, solubility, and hydrophilicity analysis. Immunogenic peptides were used to design a multi-epitope vaccine construct. Immune-modulating adjuvants and PADRE (Pan HLA-DR epitopes) sequence were added with epitopes sequence to enhance the immunogenicity. All the epitopes, adjuvants and PADRE sequence were joined by linkers. The designed vaccine constructs (VT1, VT2, VT3, and VT4) were analyzed by their physiochemical properties using different tools. Selected chimeric vaccine constructs (VT1, VT3, and VT4) were further shortlisted by their docking score with different HLA alleles. The final selected VT4 construct was docked with TLR4/MD2 complex and confirmed by molecular dynamics simulation studies. The final vaccine VT-4 construct was *in-silico* cloned in pET28a. Therefore, the designed construct VT4 may be studied to control the interaction of *P. aeruginosa* with host and infection caused by *P. aeruginosa*.

## Introduction

Hospital-based surveillance studies as well as Infectious Diseases Society of America have begun to refer nosocomial pathogens as ESKAPE pathogens that include *Enterococcus faecium, Staphylococcus aureus, Klebsiella pneumoniae, Acinetobacter baumannii, Pseudomonas aeruginosa*, and *Enterobacter species*. Different molecules such as herbal compounds^1,2^, secondary metabolites^1,3,4^ nanomaterial^5,6^ and *in-silico* designed drug^7–10^ are used to find a suitable alternative to the current antibiotics used against ESKAPE pathogen. In spite of the use of different mechanisms, to cure the ESKAPE pathogen, there is not any permanent treatment available. *P. aeruginosa* causes opportunistic nosocomial infection such as bacteremia, pneumonia and septicemia^11^. Mortality rate in bacteremia caused by *P. aeruginosa* infection is higher as compared to other Gram-negative pathogens^12^ due to the emergence of resistance in *P. aeruginosa* against most of the antibiotics^13^. Damage to the first line of defense, such as the skin or the mucous membranes, enables the colonizing bacteria to enter the bloodstream and cause septicemia^14^. Recently, there is developing attention in the various bacterial components like virulence factors for the development of vaccines and other therapies^15^. Extracellular secretory products and membrane of *P. aeruginosa* have several antigenic components that are involved in the development of immunity in the host against *P. aeruginosa* hence suitable as a vaccine targets. Major antigenic surface-associated components of *P. aeruginosa* are lipopolysaccharides (LPS) and outer membrane proteins (OMPs) because of their accessibility on the pathogen surface. In the adhesion and invasion mechanism during the host-pathogen interaction, outer membrane proteins play a major role to invade a host cell and enter in the tissue^16^. Hence, assessment of epitopes on the surface of these antigenic proteins is crucial for the development of an effective vaccine against *P. aeruginosa*^17^. LPS-based vaccines have been successfully tested in animal models as well as in clinical trials. However, the severe side effects observed in vaccinated individuals have made it necessary to develop chimeric vaccines^18,19^. Numerous vaccine candidates have been tested against this ubiquitous opportunistic pathogen. Immunization with whole cell organism^20^, killed and live attenuated vaccine^21,22^, outer membrane vesicles^23,24^, outer membrane complex^11,13,25^, flagella^26^, pilin^27^ and glycoconjugate vaccine^28^ have been suggested as efficient vaccination approach against *P. aeruginosa* due to the abundance of immunogenic components in them. Many such vaccines tested experimentally in pre-clinical trials but only a few have reached the clinical phase, and none of them has been approved for market authorization^14^.

Current immunization strategies target multiple OMPs as antigens because there is no risks of pathogen reverting back to its virulent form as well as there are few adverse effects as compared to live attenuated or killed whole cell vaccination. This bacterium rapidly acquired the resistance against multiple antibiotics, natural products, herbal products which caused the problems worldwide. Recently, subtractive genomics^29,30^ and reverse vaccinology^31,32^ are using as a potent mechanism to filter-out antigenic and immunogenic proteins that can be used as a chimeric vaccine candidate. Subtractive approach subtracts pathogen OMPs that have a role in virulence or contain the essentiality factor which is the required for survival of the *P. aeruginosa* but not present in the human host^33^. Once shortlisted, antigenic and immunogenic epitopes of all vaccine targets will be analyzed using different tools. Using these immunogenic epitopes, the chimeric vaccine can be constructed and validated.

## Materials and Methods

### Proteome of *P. aeruginosa*

To identify the chimeric vaccine candidates against pathogenic *P. aeruginosa*, we have downloaded 1191 proteomes of different strains of *P. aeruginosa* using UNIPORT server. Redundancy and non-redundancy analysis were carried out manually for 1191 *P. aeruginosa* strains. Reference proteome of *P. aeruginosa* (strain PAO1) has 5564 proteins were analyzed using different *in-silico* subtractive proteomics and reverse vaccinology approaches to determine the antigenic and immunogenic proteins/epitopes targets.

### Identification of protein location

**Figure 1:**
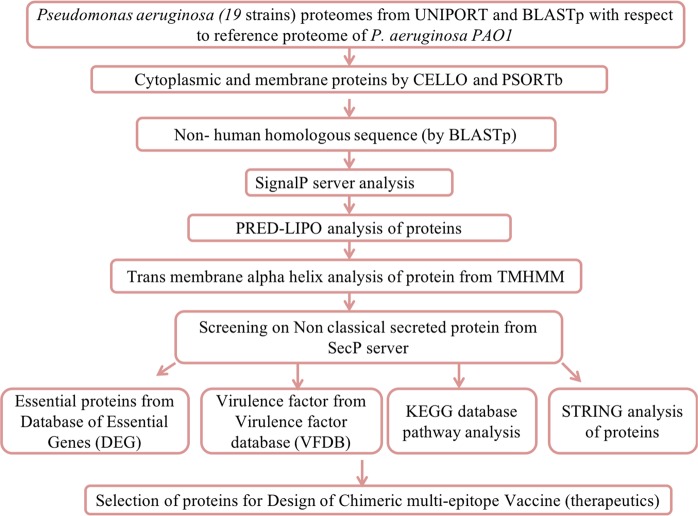
Systemic workflow of vaccine OMPs targets identification using comparative subtractive proteomics analysis.

OMPs include a functional role in bacterial pathogenesis, OM permeability barrier, efflux pumps, diffusions of nutrients, membrane integrity and in active transport. For chimeric vaccine construction, bacterial OMPs has selected because of their antigenic and immunogenic behavior^34^. OMPs that plays an essential and virulent factor role may be useful in therapeutics design. Obtaining a detailed subtractive analysis (Fig. 1) firstly we executed the cellular localization prediction of 5564 proteins using most accurate bacterial localization servers PSORTb 3.0.2^35^, and CELLO^36^. These softwares identify the localization of proteins as extracellular, outer membrane, cytoplasmic, periplasmic and inner membrane.

### Subtracting non-human homologous proteins

The vaccine candidates that are non-human homologous proteins are considered as a suitable target. This protein targets selection may minimize the probability of cross-reactivity in the host cell. For subtraction of homologous sequence between human host and pathogen, we have carried out BLASTp (https://blast.ncbi.nlm.nih.gov/Blast.cgi?PAGE=Proteins) analysis of 982 proteins at the NCBI database against the proteome of host *Homo sapiens* (taxid: 9606). The obtained hits having an E-value (expectation value) of 10^−4^ or less were considered as homologous proteins and excluded out for further analysis.

### Investigation of proteins exported to periplasmic space

Using different servers (PSORTb 3.0.2 and CELLO), we have confirmed the proteins cellular location in the cell. Secretary nature of 918 non-human homologous proteins, were predicted using SignalP 4.0^37^. Proteins being exported from the inner membrane to periplasm following the sec pathway (secretory pathway) were predicted by identifying the presence of N-terminal signal sequences. The positive signal contained proteins were shortlisted to carry out further analysis.

### Lipoprotein signal peptide detection

In bacteria, lipoproteins are determined as antigenic and immunogenic targets. To predict potential lipoproteins among the obtained exported proteins, we were used highly sensitive and specific PRED-LIPO software^38^. Proteins that contained the positive PRED-LIPO signal were considered as good antigenic targets. Shortlisted 482 secretary proteins were analyzed by PRED-LIPO program. 82 proteins were found to be lipoproteins and were separated from the list and the remaining 400 proteins were considered for further experiment. Out of these 400 proteins, 101 proteins are located in OMPs and extra-cellular proteins (ECPs) hence considered for further analysis.

### Identification of trans-membrane alpha-helices

The trans-membrane integral inner membrane proteins (IMPs) are the plasma membrane-spanning proteins and contain α-helices. To subtract those proteins that are being exported and getting embedded in the plasma membrane, the 101 obtained proteins were tested in TMHMM (Trans-membrane helices hidden Markov model for topology prediction) 2.0^39^ server. The *P. aeruginosa* exported proteins that contain more than one TM α-helix with the standard settings were considered as integral IMPs. Non-IMPs proteins (<1 TM α-helix) were selected for further experiments.

### Proteins follow signal peptide-independent secretion pathway

Classical pathway transports protein from cytosol to periplasm while the non-classical pathway transports protein from periplasm to extracellular region. Non-classical pathway secreted proteins do not contain the signal peptides but still found in the bacterial extracellular region. With the help of SecretomeP 2.0 program^40^, 83 proteins were excluded due to its non-classical secreted extracellular proteins. The proteins with a SecP score of 0.5 and above were considered to be non-classically secreted. Finalized OMPs were BLAST against the 10 randomly selected non-redundant proteome using Ensembl database.

### Identification of OMPs with an essential role

The database of essential genes (DEG) includes experimentally identified essential proteins which determined the pathogen essential proteins^41^. In DEG, BLASTp search was performed with a cut-off of 1e^−04^ E-value, and a bit score of 100, as well as the BLOSUM62 matrix that shortlisted fifteen OMPs of *P. aeruginosa*.

### Identification of virulence-associated OMPs

Virulence factors associated with OMPs have a major role in host-pathogen interaction and modulate host-defense mechanism. VFDB database is used for the identification of virulence-associated OMPs^42^. *P. aeruginosa* identified 15OMPs were subjected to BLASTp against VFDB protein core dataset (R1) with E-value was 1e^−04^ and default cut-off bit score >100. All 15 OMPs were predicted to be virulent.

### Identification of proteins involved in pathogen-specific pathways

Kyoto encyclopedia of gene and genome (KEGG) pathway database^43^ explains the role of proteins in different metabolic pathways. To identify the unique and common pathways between the human host and *P. aeruginosa*, we have compared all enlisted metabolic pathways manually. The 15 OMPs were segregated based on their role in pathogen-specific (unique), and common (pathogen & host both) pathways. We have also identified the functional role of these 15 OMPs via pathways dependent or pathway independent analysis.

### Antigenicity prediction of OMPs

**Figure 2:**
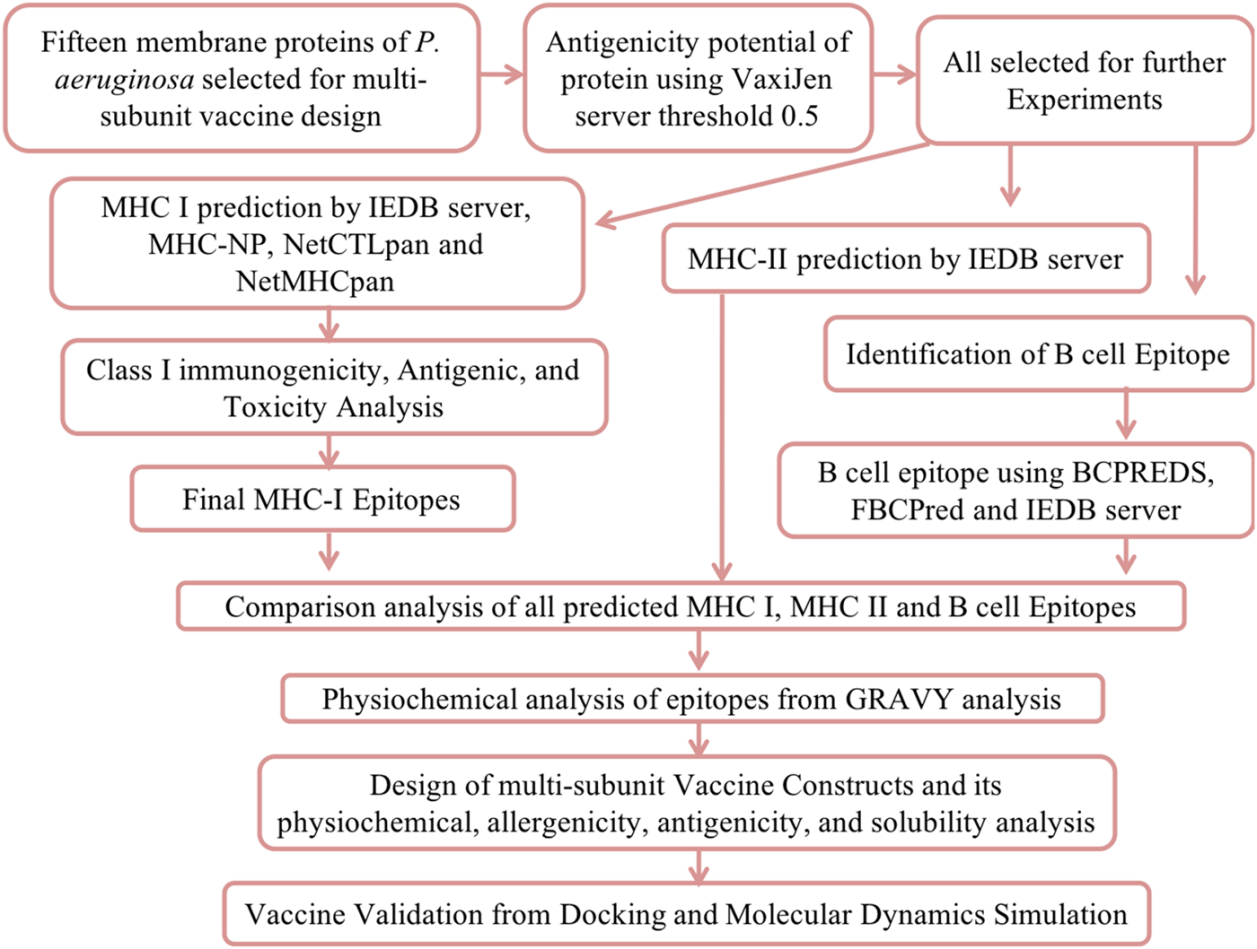
Illustration of chimeric multi-epitope vaccine design using reverse vaccine workflow.

As discussed above, comparative subtractive proteomics approach filtered-out 15 virulent OMPs out of 5564 proteins of *P. aeruginosa*. These 15 OMPs were considered as potential targets for chimeric vaccine design. Antigenicity of the virulent OMPs can enhance the immune response in the host cell; hence, we performed reverse vaccinology analysis outlined in Fig. 2. Firstly, we carried out antigenicity analysis using VaxiJen server^44^. The proteins that contained the score value > 0.5 were considered for afterward analysis as an antigenic OMPs.

### Protein-protein interaction network analysis

A chimeric vaccine against OMPs will affect the different protein expressions in the bacterial cell. These vaccines may affect the intra-species OMPs interacting partners that resulted reduce the pathogenesis of *P. aeruginosa* infection. All OMPs protein-protein intra-species interaction analysis was executed to find out the proteins network for identified OMPs using protein interaction database STRING^45^.

### T cell MHC Class I epitope prediction

Antigenic 15 virulent OMPs were selected for the reverse vaccinology analysis. We have performed different T-cell epitope analysis for potent epitopes identification, using four independent servers like IEDB MHC-I prediction server^46^, MHC-NP^47^, NetCTLpan1.1^48^, and NetMHCpan3.0^49^. In the host cell proteasomal complex, pathogenic proteins naturally proceed into epitopes. The immune response against the pathogen is initiated by T-cell that interacts with MHC class I represented epitopes. The IEDB (http://tools.immuneepitope.org/processing/) MHC-I processing predictions server identify epitopes that can interact with MHC I molecules. We have selected a MHC allele reference file that contains 27 different alleles that cover more than 97% of the world population. Other parameters of prediction tools were set to default. The MHC-NP server (http://tools.immuneepitope.org/mhcnp/) used the MHC elution experiment to predict the probability that a given peptide is naturally processed. The NetCTLpan1.1 (http://www.cbs.dtu.dk/services/NetCTLpan/) server is predicted proteins cytotoxic lymphocyte (CTL) epitopes. This server uses weight matrix, artificial neural networks (ANN), and TAP transport efficiency to predict peptides that have the potential for MHC-I binding, and proteasomal C terminal cleavage site. NetMHCpan3.0 (http://www.cbs.dtu.dk/services/NetMHCpan/) server using ANN predicts the ability of peptide-MHC class I binding. All 15 OMPs were analyzed by all four prediction methods individually. The top predicted epitopes from all the proteins were selected. Only those epitopes were selected for the further analysis presented in all four servers.

### Class I immunogenicity prediction

IEDB server (http://tools.immuneepitope.org/immunogenicity/), a MHC I immunogenicity prediction tool was applied to identify MHC-Peptide complexes with potential immunogenicity in the infected host cell^50^. The shortlisted epitopes were analyzed using this server with default parameters. The epitopes that gained a positive immunogenicity score were selected for further analysis.

### T-cell MHC Class II epitope prediction

IEDB, MHC Class II prediction server is used to identify T-cell epitopes that can bind to MHC class II molecules^51^. The consensus (stabilization matrix alignment, and average relative binding matrix) prediction approach used to identify the potent MHC II binding epitopes.

### Analysis of antigenic and toxicity behavior of epitopes

Obtained immunogenic epitopes (MHC I and MHC II) were analyzed for their antigenic and toxicity behavior. Both analyses were carried out using VaxiJen version 2.0^44^ and ToxinPred tool^52^ respectively. VaxiJen server (>0.5) predicts the antigenic behavior of epitopes by their physicochemical behavior. In the host cell, the specific immune response induced that will target only bacterial cell or also host cell was analyzed by the ToxinPred tool.

### B-cell epitope prediction

To initiate humoral immunity, epitopes should be recognized by B lymphocytes. B-cell epitopes were predicted by the different server like BCPREDS^53^, the FBCPred server^54^ and IEDB server^55^. BCPRED and FBCPred cut-off score used in the present study is >0.8. Epitopes that predicted by all three servers were chosen for further study.

### Comparison of predicted epitopes to select final epitopes for vaccine design

Final chimeric vaccine sequence was designed using B-cell epitopes, MHC-I epitopes, and MHC-II epitopes after manual comparative analysis. Overlapping epitopes sequences were merged and used to constructs the final vaccine.

### Hydropathy analysis of epitopes

For chimeric OMPs vaccine, all epitopes should have the hydrophilic nature (present on the surface) otherwise the epitopes will not be able to initiate the immune response in the host cell. Hydrophilic epitopes of OMPs are present on the surface of bacterium while hydrophobic epitopes are spanning in the bacterial membrane. GRAVY score analysis of epitopes was done using the ProtParam tool. The GRAVY (grand average of hydropathy) value of protein is calculated as the total of all the amino acids hydropathy values, divided by the number of amino acid residues in a given protein. A positive GRAVY value indicates that the protein is hydrophobic whereas a negative value indicates that protein contains hydrophilic properties.

### MHC restricted alleles using cluster analysis

Using IEDB analysis, we have shortlisted the MHC class I and class II-restricted epitopes. MHCcluster v2.0 server was the additional crosscheck of identified epitopes, to confirm the prediction^56^. The server describes peptides and HLA functional relationship in the form of a static heat map and graphical tree.

### Construction of design model vaccine

To design chimeric vaccine targeted to OMPs, we were joined all the selected OMPs epitopes (HTL, CTL and B epitopes) using amino acid linkers (HEYGAEALERAG and GGGS linkers). Immunogenicity of constructs was enhanced by the addition of different adjuvants using ‘EAAAK’ linkers at both terminus (N and C). These adjuvant were 50 s ribosomal L7/L12 protein^57^, beta-defensin^58^, HBHA protein (*M. tuberculosis*, accession no. AGV15514.1), and HBHA conserved sequence^59^ respectively. To improve the vaccine efficacy and potency non-natural pan DR (PADRE) 13 amino acid epitope (AKVAAWTLKAAAC) that induce CD4+ T-cells were also combined along with the adjuvants. Alexander *et al*. also explain that if PADRE glycoconjugates (i.e KXVAAWTLKAAZC, where X is l-cyclohexylalanine & Z is aminocaproic acid) is used then it will further enhance the IgG production^60^. Polymorphism of HLA-DR molecules in the worldwide population is overcome by PADRE sequence that has 100-fold more potency than the universal T helper epitopes. It was found that vaccine construct with PADRE sequence exhibited better CTL responses than a vaccine without it^61^. All adjuvant proteins are agonist to different TLR complexes that polarizes CTL responses which have a robust immuno-stimulatory effect^62^.

### Allergenicity, antigenicity and solubility prediction of design vaccine constructs

For a selection of the suitable vaccine constructs, all four vaccine constructs (VT1, VT2, VT3, and VT4) were analyzed using on the basis of their antigenicity, allergenicity and soluble prediction methods. For the allergenic behavior of vaccine, the constructs were analyzed by the AlgPred server^63^. Antigenicity of vaccine constructs was predicted using two servers: ANTIGENpro^64^ and VaxiJen 2.0^44^ server. Vaccine constructs solubility and corresponding probability (≥0.5) were predicted using SOLpro server^64^.

### Physicochemical behavior analysis of vaccine constructs

Vaccine constructs physiochemical properties were characterized using Expasy ProtParam server^65^. This server is widely used for determining the number of amino acids, PI values, molecular weight, aliphatic index, instability index, and hydropathicity GRAVY values. Instability index of protein predicts proteins stability (<40). Aliphatic index value explains about vaccines thermostability. GRAVY explains the proteins hydrophilic or hydrophobic nature.

### Prediction of the secondary structure

Various vaccine constructs were used for predicting its secondary structure components using PSIPRED v3.3 program^66^. PSIPRED 3.2 server accuracy is 81.6% which predicts the proteins alpha and beta helix and coil structure.

### Molecular Docking study and Molecular Dynamics Simulation

Finalized three vaccine constructs (VT1, VT3, and VT4) were intensively modeled using the Phre2 online tool. Different HLA alleles PDB ID i.e. DRB1*03:01(1A6A), DRB1*15:01(1BX2), DRB3*02:02(3C5J), DRB5*01:01(1H1S), HLA-B*44:03(1SYS), HLA-B*53:01(1A1M), HLA-B*15:01(1XR8), HLA-B*39:01(4O2E), HLA-B*58:01(5IM7) and HLA-B*35:01(1ZSD) were downloaded from protein data bank (RCSB). With the help of PatchDock server, docking of three vaccine constructs was performed with 10 different HLA alleles. Similarly, we also docked the vaccine construct (VT4) with TLR 4/MD2 complex (PDB ID 3FXI) with the help of PatchDock^67^. The 10 best solutions of PatchDock result were further refinement using FireDock. Docking of the vaccine construct (VT-4) with TLR 4/MD2 complex was also performed using ClusPro^68^. VT4-TLR4 complex molecular dynamics simulation was performed using Gromacs v5.1.2 as the published method^69^.

### Codon optimization of design vaccine construct VT-4 and *In-silico* cloning

Java Codon Adaptation Tool (JCAT) was developed to improve heterologous protein production in *E. coli* host strain^70^. Vaccine VT4 construct was reverse translated, and subsequently adapted for codon usage to *E. coli*. The prokaryotic ribosome binding sites, rho-independent transcription terminators, and few restriction sites were avoided. Gene sequence of final design vaccine construct VT-4 was cloned in *E. coli* pET28a vector, by Snapgene tool^71^ that make sure vaccine construct expression.

## Results

### Comparative subtractive proteomic approach shortlisted *P. aeruginosa* strains

With the help of UNIPORT server, proteomes of different strains of *Pseudomonas* genospecies were downloaded that contains the list of 1224 strains. From 1224 different *Pseudomonas* strains, we have filtered out the 1191 strains of *P. aeruginosa* for further analysis. Redundancy and non-redundancy analysis were carried out manually for the proteome of 1191 *P. aeruginosa* strains. During comparison, 11 different groups of non-redundant strains were made. Nine strains of *P. aeruginosa* did not show any redundancy with the other *Pseudomonas* strains. Therefore, a total number of 20 (11 + 9) (Supplementary Table ST-1) strains of *Pseudomonas* were selected for further analysis. Proteome of *P. aeruginosa* PAO1 strain is considered as a reference proteome in the present study.

### Identification of subcellular localization of the proteins

Vaccine design using subtractive proteome analysis against the pathogen could minimize time, and resources for developing the therapeutic agent that have large targets. To achieve the potent proteins for vaccine construct, firstly, we have filtered all 5564 proteins (of reference proteome) on the basis of their sub-cellular localization. With the help of CELLO and PSORTb tool, we have filtered 982 different proteins out of 5564 proteins of *P. aeruginosa* PAO1. These 982 proteins localization is in the periplasm, the extracellular and outer membrane of the bacterial cell.

### Identification of bacterial proteins that are non-human homologous

To exclude out the cross-reactivity of vaccine with the human host cell, the potent vaccine should be designed against non-human homologous proteins. We found 918 non-human homologous proteins out of 982 proteins using BLASTp. These non-human homologous bacterial proteins were lead to vaccine construct that only interacts with the proteins of *P. aeruginosa*.

### Signal peptide detection

The exported proteins are secreted out across the inner membrane and enter the periplasmic space. With the help of a positive signal in SignalP server, 482 *P. aeuginosa* proteins (out of 918 proteins) were selected as exported proteins. The exported proteins have more chance to be transported and located in the bacterial surface that might be a better target for vaccine design. Therefore, proteins with exported capacity were selected for further experiments.

### Lipoproteins of *P. aeruginosa*

With the help of acyl chain, bacterial lipoproteins are located in the outer membrane, inner membrane, and periplasm. With using PRED_LIPO program, we successfully identified 82 *P. aeruginosa* proteins as a lipoprotein. These lipoproteins can also be considered as potent antigenic targets, but in the present study, we are targeting the OMPs. Therefore, for designing chimeric vaccine constructs, we have excluded out these 82 lipoproteins and rest 400 proteins were considered in further experiments.

### TM α-helices containing proteins

In bacteria, secretory exported proteins have different locations. Some of the proteins located in the periplasm, some of them embedded into the outer membrane and some of them embedded in the periplasmic side of the inner membrane. These IMPs contain TM hydrophobic α-helices that act as an internal signal and helps in getting them embedded in the membrane. Out of 400 proteins, we only considered 101 proteins which are located in the OMPs. Using TMHMM server, we have found 18 *P. aeruginosa* proteins that contain more than one TM α-helices. These 18 proteins are considered to be inner membrane proteins and exclude out. Finally, filtered 83 were considered for further analysis.

### Selection of OMPs after elimination of the non-classical secretory proteins

Classical secretion pathway involved signal peptide while non-classical secretion system does not involve signal peptide sequence. To eliminate the non-classical secretory proteins, we have used SecretomeP 2.0 program. In SecretomeP 2.0 server, we have analyzed the 83 OMPs proteins of *P. aeruginosa*. In secP server, proteins that scored > 0.5 were considered as non-classical secretory proteins. We found 68 out of 83 *P. aeruginosa* proteins as non-classical secretory proteins. These proteins may be located in the extracellular region of the bacterial cell hence excluded from further analysis. This analysis leads to the selection of the final 15 unique OMPs proteins (Table 1). BLAST using Ensembl genome database showed that all the 15 OMPs have sequence similarity with the 10 randomly selected non-redundant proteomes.

**Table 1:** Characterization of outer membrane proteins (OMPs) using different server.

S.No.	Protein name	Uniport ID^1^	Mol. Wt^2^	Cellular component^3^	Virulence factor^4^	DEG analysis^5^	Function^6^	Pathway^7^	Antigenicity^8^
1	Type II secretion system protein D	P35818	69.953	Outer membrane	Yes	No	Identical protein binding, protein transporter activity, Type II secretion system	Bacterial secretion system,biofilm formation	0.6024
2	Uncharacterized protein	Q9HU51	47.291	Outer membrane	Yes	Yes	—	—	0.548
3	Uncharacterized protein	Q9I3A9	78.959	Outer membrane	Yes	No	—	iron complex outermembrane recepter protein	0.5991
4	Uncharacterized protein	Q9I6G3	70.362	Outer membrane	Yes	No	Aminopeptidase acitivity, autotransporter activity, cell motility, pathogensis	—	0.6066
5	Pyroglutatmate porin OpdO	Q9I202	44.302	Outer membrane	Yes	No	—	—	0.7592
6	Probable porin	Q9I4U9	46.520	Outer membrane	Yes	No	—	—	0.7357
7	Probable TonB-dependent receptor	Q9I2I2	72.261	Outer membrane	Yes	No	—	cirA, cfrA, hmuR; outer membrane receptor for ferrienterochelin and colicins	0.7801
8	Type III secretion outer membrane protein PscC	Q9I319	65.734	Outer membrane	Yes	No	Protein transporter activity, protein type III secretion system	Bacterial secretion system, pathogenicity	0.5844
9	Uncharacterized protein	Q9I792	63.191	Outer membrane	Yes	No	Protein transport	Infectious disease	0.726
10	Outer membrane protein CzcC	Q9I0W0	46.794	Outer membrane	Yes	No	Efflux transmembrane transporter activity	czcC; outer membrane protein, cobalt-zinc-cadmium efflux system	0.6038
11	Probable outer membrane protein	Q9HVJ4	55.227	Outer membrane	Yes	No	Efflux transmembrane transporter activity	—	0.6472
12	Probable outer membrane protein	Q9HYK0	49.935	Outer membrane	Yes	No	Efflux transmembrane transporter activity	Metalloprotease transport system, outer membrane protein, protease secretion system	0.6742
13	OpdB proline porin	Q9I0E2	47.318	Outer membrane	Yes	No	—	—	0.7906
14	Patatin-like protein, PlpD	Q9HYQ6	80.898	Outer membrane	Yes	No	Lipase activity, lipid catabolic process, type V secretion system	—	0.5043
15	Probable tonB-dependent receptor	Q9I473	67.568	Outer membrane	Yes	No	Cobalamin transporting ATPase activity	Signaling and cellular process(vitamin B12 transporter)	0.6877

All data were analyzed using different server 1,6 = uniport server, 2 = ProtParam server, 3 = CELLO,PSORTb, 4 = VFDB database, 5 = DEG database,  7 = KEGG database,  8 = VaxiJen server.

### OMPs have an essential protein of *P. aeruginosa*

Essential proteins as vaccine targets will cause the major effect on the bacterium, Hence, we have analyzed 15 OMPs proteins using DEG server (Table 1). Out of 15 proteins, one protein Q9HU51; uncharacterized protein is considered essential for the survival of *P. aeruginosa* strain.

### All selected OMPs have a role in the virulence of *P. aeruginosa*

A chimeric vaccine against the virulent factor associated OMPs will enhance the potency and efficiency of the protein. The VFDB database result (Table 1) showed that all 15 proteins are involved in the virulence of *P. aeruginosa*. Hence, these proteins are also considered as an essential target to inhibit the pathogenesis of *P. aeruginosa*.

### Pathogen-specific OMPs have been selected

*P. aeruginosa* and human host metabolic pathways (present in KEGG Database) manual comparison have done to find the shortlisted outer membrane pathogenic proteins role in the different pathways. This result identifies 42 unique pathogen pathways (Supplementary Table ST-2) with respect to *P. aeruginosa* and the remaining 79 pathways were common and found in both pathogen and host. Eight out of fifteen OMPs found to posse’s metabolic pathways dependent and 7 remaining proteins were metabolic pathway independent proteins (Table 1).

### Outer membrane proteins are antigenic

From the subtractive proteome analysis, we have filtered out 15 OMPs that may use as promiscuous vaccine targets. We analyzed the proteins antigenicity using VaxiJen web server that found all are the potent antigenic OMPs (Table 1). All 15 proteins were identified with the prediction score ranging from  0.5043 to 0.7906 .

### Selected proteins showed intra-species protein-protein interaction

STRING predictions of fifteen OMPs of *P. aeruginosa* showed the different intra-species interaction between the proteins. With the help of all interaction study, we might understand the protein networking of 15 OMPs. A chimeric vaccine against these 15 OMPs may affect all interacting network proteins that further enhance the efficacy of the chimeric vaccine. All proteins networking is shown in Supplementary Figure SF 1.

### Selected OMPs have MHC I epitopes

T-cell epitopes of 15 OMPs were used for MHC-I binding prediction by IEDB server. The epitopes with higher affinity (IC < 50 nM) and good percentile rank (≤0.2) were selected for analysis. IEDB MHC1, MHC-NP, netCTLpan1.1, and netMHCpan3.0 B cell epitope analysis identified the potent epitopes. From all servers, we have identified 221 epitopes (Supplementary Table ST-3) in the 15 antigenic OMPs. To improve accuracy, we have filtered epitopes that were predicted by all four tools. In total, 80 common epitopes out of 221 epitopes were selected from these four servers.

### Selected MHC I epitopes are Immunogenic

Selected 80 MHC class I epitopes from different servers were analyzed for a class I immunogenicity and toxicity. A high immunogenicity score showed high potency to stimulate naive T cells and also induce cell-mediated immunity. In IEDB immunogenicity prediction, 80 epitopes gained a score range from 2.9388 to −0.0193 (Supplementary Table ST-3). One protein i.e. uncharacterized protein (Q9HU51) identified potent epitopes did not show the positive values score hence excluded from further analysis. In this analysis, 50 epitopes of 14 different OMPs contained the positive value score were selected for further analysis (Table 2).

**Table 2:** Identification of MHC I epitopes using four different server and prediction of class I immunogenicity.

S.No.	Protein ID, name	start	end	Epitope	IEDB (MHC I binding)	MHC-NP	NetCTLpan	NetMHC	Class I immunogenicity
1	Q9I6G3, Uncharacterized protein	193	201	VSNPALGAY	HLA-A*30:02(0.1)	HLA-B*53:01 (0.681)	HLA-A*01:01(0.20), HLA-B*58:01(0.80), HLA-B*15:01(0.40)	HLA-A*26:01(0.3064)	0.06586
		467	475	LQAAGGPVL	HLA-B*15:01(0.2)	HLA-B*53:01(0.6489)	HLA-B*39:01(0.15), HLA-B*40:01(0.80)	HLA-B*39:01(0.0973)	0.13173
		66	74	QTSRQDFTW	HLA-B*58:01(0.2)	HLA-B*53:01(0.7424)	HLA-B*58:01(0.05)	HLA-B*58:01(0.0076)	0.02794
		542	550	LEASLGWRL	HLA-B*40:01(0.25)	HLA-B*44:03(0.2787)	HLA-B*40:01(0.10)	HLA-B*40:01(0.0556)	0.08451
		66	74	QTSRQDFTW	HLA-B*57:01(0.25)	HLA-B*53:01(0.7424)	HLA-B*58:01(0.05)	HLA-B*58:01(0.0076)	0.02794
2	Q9HVJ4, Probable outer membrane protein	134	142	RTADEAGRY	HLA-A*30:02(0.1)	HLA-B*44:03(0.6287)	HLA-A*01:01(0.05), HLA-A*26:01(0.30)	HLA-A*01:01(0.0704)	0.22819
		119	127	GEYGTRFSL	HLA-B*40:01(0.15)	HLA-B*44:03(0.2256)	HLA-B*39:01(0.40), HLA-B*40:01(0.01)	HLA-B*39:01(0.1811)	0.12156
3	Q9I0E2, OpdB proline porin	173	181	IQAGRFTAF	HLAB*15:01(0.1)	HLA-B*53:01(0.7975)	HLA-B*08:01(0.80), HLA-B*15:01(0.05)	HLA-B*15:01(0.0070), HLA-B*08:01(0.3041)	0.26302
		137	146	GEMTVETPVF	HLA-B*44:02(0.25)	HLA-B*57:01(0.7617)	HLA-B*40:01(0.20)	HLA-B*40:01(0.1780)	0.17106
4	Q9HYK0, Probable outer membrane protein	356	364	LAKVRAYEM	HLA-B*08:01(0.2)	HLA-B*53:01(0.3206)	HLA-B*08:01(0.30)	HLA-B*08:01(0.0303)	0.11415
		199	207	RAAAARRTL	HLA-B*07:02(0.2)	HLA-B*57:01(0.7068)	HLA-B*07:02(0.20)	HLA-B*07:02 (0.0826)	0.20525
		222	230	APIERFPAL	HLA-B*07:02(0.2)	HLA-B*07:02(0.5309)	HLA-B*07:02(0.05), HLA-B*08:01(0.05), HLA-B*39:01(0.30)	HLA-B*07:02(0.0083)	0.31805
5	Q9HYQ6, Patatin-like protein, PlpD	470	478	RYFVAPFLF	HLA-A*23:01(0.1), HLA-A*24:02(0.1)	HLA-B*53:01(0.4128)	HLA-A*24:02(0.01)	HLA-A*24:02(0.2738)	0.19952
		476	484	FLFHEAQNV	HLA-A*02:01(0.2)	HLA-A*02:01(0.2671)	HLA-A*02:01(0.10)	HLA-A*02:01(0.0126)	0.10334
		181	189	LPQAIRASM	HLA-B*07:02(0.2)	HLA-B*07:02(0.3453)	HLA-B*08:01(0.80), HLA-B*07:02(0.05)	HLA-B*07:02(0.0078)	0.11645
		476	484	FLFHEAQNV	HLA-A*02:03(0.25)	HLA-A*02:01(0.2671)	HLA-A*02:01(0.10)	HLA-A*02:01(0.0126)	0.10334
6	Q9I0W0, Outer membrane protein CzcC	334	342	LLRLRSEAV	HLA-B*08:01(0.2)	HLA-A*02:01(0.0807)	HLA-B*08:01(0.10)	HLA-B*08:01(0.0564)	0.00767
		294	302	GERVNLIGL	HLA-B*40:01(0.25)	HLA-B*53:01(0.0519)	HLA-B*40:01(0.40)	HLA-B*40:01(0.1368)	0.17372
7	Q9I2I2, Probable TonB-dependent receptor	14	23	RLARAVPFLY	HLA-A*30:02(0.1)	HLA-B*57:01(0.8863)	HLA-A*02:01(0.30)	HLA-A*03:01(0.3228)	0.22496
		522	530	LELGGGVDL	HLA-B*40:01(0.15)	HLA-B*53:01(0.5935)	HLA-B*40:01(0.10)	HLA-B*40:01(0.0361)	0.1432
		68	77	RPVRDLQEAL	HLA-B*07:02(0.15)	HLA-B*57:01(0.9091)	HLA-B*07:02(0.80)	HLA-B*07:02(0.0263)	0.0746
		304	312	RTYRNRLER	HLAA*31:01(0.15)	HLA-B*57:01(0.1241)	HLA-A*03:01(0.30)	HLA-A*03:01(0.1019)	0.14244
		162	170	ITRRATDTW	HLA-B*57:01(0.15)	HLA-B*53:01(0.6409)	HLA-B*58:01(0.05)	HLA-B*58:01(0.0158)	0.18492
		404	412	WESSPRLYL	HLA-B*40:01(0.2)	HLA-B*53:01(0.191)	HLA-B*40:01(0.10)	HLA-B*39:01(0.3844)	0.19377
		595	603	YALPAYSLW	HLA-B*58:01(0.2), HLA-B*57:01(0.25)	HLA-B*53:01(0.8414)	HLA-A*24:02(0.40)	HLA-B*58:01(0.0054)	0.12624
		49	57	KLRDAPASV	HLA-A*02:03(0.25)	HLA-A*02:01(0.7401)	HLA-A*02:01(0.80)	HLA-A*02:01(0.2252)	0.00314
		288	296	WSLAHNGQW	HLA-B*57:01(0.25)	HLA-B*53:01(0.798)	HLA-B*58:01(0.20)	HLA-B*58:01(0.0741)	0.0221
8	Q9I4U9, Probable porin	325	333	RSWQLRYDY	HLA-A*30:02(0.15)	HLA-B*44:03(0.598)	HLA-B*58:01(0.40)	HLA-B*58:01 (0.2830)	0.0031
		64	72	YESGYTEGL	HLA-B*40:01(0.15)	HLA-B*53:01(0.4653)	HLA-B*39:01(0.80)	HLA-B*39:01(0.2399)	0.11764
		273	281	ALNALFTYR	HLA-A*31:01(0.15)	HLA-B*44:03(0.7219)	HLA-A*03:01(0.40)	HLA-A*03:01(0.1388)	0.16727
		134	142	MPRLPVVQF	HLA-B*07:02(0.2)	HLA-B*35:01(0.8115)	HLA-B*07:02(0.05)	HLA-B*07:02(0.0156)	0.00086
		308	316	PYLVNFVQI	HLA-A*23:01(0.25)	HLA-B*53:01(0.115)	HLA-A*24:02 (0.15)	HLA-A*24:02(0.1399)	0.109
9	Q9I319, Type III secretion outer mem-brane protein PscC	120	128	RALTAAGIW	HLA-B*57:01(0.15)	HLA-B*53:01(0.7294)	HLA-B*58:01(0.20)	HLA-B*58:01(0.0267)	0.21675
		200	209	IEAPGIASIL	HLA-B*40:01(0.2)	HLA-B*57:01(0.9009)	HLA-B*40:01(0.30)	HLA-B*40:01(0.0835)	0.13911
10	Q9I473, Probable tonB-dependent receptor	521	529	LPRRARRMF	HLA-B*07:02(0.1)	HLA-B*57:01(0.407)	HLA-B*08:01(0.80)	HLA-B*07:02(0.1439)	0.09678
		93	101	TESDHVLVL	HLA-B*40:01(0.15)	HLA-B*53:01(0.6523)	HLA-B*39:01(0.80)	HLA-B*39:01(0.1398)	0.05374
		401	409	TVSYGTAFK	HLA-A*68:01(0.15), HLA-A*11:01(0.2)	HLA-B*53:01(0.2427)	HLA-B*58:01(0.80)	HLA-A*03:01 (0.0948)	0.11354
		590	598	FGADHETAY	HLA-B*35:01(0.2)	HLA-B*53:01(0.9534)	HLA-A*26:01(0.80)	HLA-B*15:01(0.2933)	0.21639
11	Q9I792, Uncharacterized protein	554	562	IYFRVDAFF	HLA-A*23:01(0.15)	HLA-B*53:01(0.4703)	HLA-A*24:02(0.01)	HLA-A*24:02(0.0180)	0.25258
		529	537	RYFAASVGF	HLAA*23:01(0.25)	HLA-B*44:03(0.1984)	HLA-A*24:02 (0.10)	HLA-A*24:02(0.0167)	0.01438
12	P35818, Type II secretion system protein D	49	57	QEAHWTINL	HLA-B*40:01(0.25)	HLA-B*44:03(0.105)	HLA-B*40:01(0.30)	HLA-B*40:01 (0.1275)	0.40603
13	Q9I3A9, Uncharacterized protein	694	702	LYFSAEVTF	HLA-A*23:01(0.15)	HLA-B*53:01(0.8723)	HLA-A*24:02 (0.10)	HLA-A*24:02(0.0174)	0.0614
		530	538	REIGYNGFF	HLA-B*40:01(0.2)	HLA-B*44:03(0.4882)	HLA-B*40:01(0.10)	HLA-B*40:01(0.1365)	0.16461
		458	466	SPRLAVNYL	HLA-B*07:02(0.2)	HLA-B*07:02(0.5578)	HLA-B*07:02(0.05)	HLA-B*07:02(0.0087)	0.07498
		619	627	GSAGWMHDW	HLA-B*57:01(0.2)	HLA-B*53:01(0.8763)	HLA-B*58:01(0.15)	HLA-B*58:01(0.0296)	0.13746
14	Q9I202, Pyroglutatmate porin OpdO	319	327	RSWQLRYDY	HLA-A*30:02(0.15)	HLA-B*44:03(0.598)	HLA-A*01:01(0.80)	HLA-B*58:01(0.2830)	0.0031
		255	263	SEDGGFREL	HLA-B*40:01(0.2)	HLA-B*53:01(0.8133)	HLA-B*39:01(0.30)	HLA-B*39:01 (0.2621)	0.28668
		129	137	LLKVGALHF	HLA-B*15:01(0.2)	HLA-B*53:01(0.7471)	HLA-B*15:01(0.05)	HLA-B*15:01(0.0899)	0.05091
		4	12	TPRLAAALL	HLA-B*07:02(0.2)	HLA-B*07:02(0.4789)	HLA-B*07:02(0.20)	HLA-B*07:02(0.0177)	0.10711
		150	158	LPELFRGAL	HLA-B*07:02(0.2)	HLA-B*44:03(0.0961)	HLA-B*07:02 (0.15)	HLA-B*07:02 (0.0367)	0.23552

### Selection of MHC-II epitopes in OMPs for a chimeric vaccine design

MHC-II binding predictions of all 15 OMPs were subjected using IEDB server. On the basis of percentile rank as well as IC50 value (<50 nM), 41 epitopes (Supplementary Table ST-5) were selected for afterwards analysis. MHC II prediction showed that thirteen out of 15 OMPs showed low percentile rank and higher affinity epitopes. Two OMPs (Q9I0W0; Outer membrane protein CzcC, and Q9I0E2; OpdB proline porin) did not show a good percentile for MHC II class epitopes.

### Toxicity and antigenicity prediction of MHC class I and MHC class II epitopes

Epitopes induced cross-reactivity in the host tissue was identified using the ToxinPred server. The 50 MHC I epitopes and 41 MHC II epitopes were found to be non-toxic (Supplementary Tables ST-4 and ST-5). Antigenicity of potent MHC I epitope and MHC II epitopes were analyzed using VaxiJen web server. MHC I epitopes of protein (Q9I319, type III secretion outer membrane protein PscC) and MHC II epitopes of protein (Q9I319, Type III secretion outer membrane protein PscC and Q9HU51, uncharacterized protein) antigenicity value was less than 0.5, so these proteins epitopes were excluded as non-antigenic proteins. The result showed a total of 52 (30 + 22) epitopes contained the potent antigenicity. These 52 (30 + 22) (Supplementary Table ST-4 and Table 3) epitopes of MHC class I and MHC class II were selected.

**Table 3:** MHC II epitpoe analysis using IEDB server and their toxicity and antigenicity analysis.

S.No.	Name	Uniport ID	Start	End	Epitope	IEDB (percentile rank)	Toxicity	Antigencity
1	Type II secretion system protein D	P35818	74	88	GETFVVDPRVKGQVS	HLA-DRB1*11:01(0.19)	Non- toxin	0.7776
2	Uncharacterized protein	Q9I3A9	665	679	ALELAGVLQQRLDDQ	HLA-DRB4*01:01(0.13)	Non- toxin	0.8109
3	Uncharacterized protein	Q9I6G3	471	485	GGPVLLDQRLSGDTS	HLA-DRB1*03:01(0.05)	Non- toxin	0.7245
4	Pyroglutatmate porin OpdO	Q9I202	266	280	RAFGALFSLRLGAHA	HLA-DRB1*09:01(0.01)	Non- toxin	1.0951
			182	196	SSDYQVFSANRIGGR	HLA-DRB1*07:01(0.24)	Non- toxin	0.6490
5	Probable porin	Q9I4U9	58	72	QGFLLRYESGYTEGL	HLA-DRB1*15:01(0.14)	Non- toxin	0.7220
6	Probable TonB-dependent receptor	Q9I2I2	431	445	KAPSLKQLSPEYAAV	HLA-DRB1*09:01(0.03)	Non- toxin	0.6907
			10	24	LPPLRLARAVPFLYL	HLA-DRB3*02:02(0.06)	Non- toxin	1.0610
			536	550	WELNYTYLDARNRTA	HLA-DRB5*01:01(0.15)	Non- toxin	0.9702
7	Uncharacterized protein	Q9I792	519	533	GNAIELDARGRYFAA	HLA-DRB1*03:01(0.02)	Non- toxin	0.6998
8	Probable outer membrane protein	Q9HVJ4	204	218	DAKYRAGAAALSDRL	HLA-DRB1*09:01(0.02)	Non- toxin	0.7788
			163	177	ATLQNTFALAAQAYY	HLA-DQA1*01:02/DQB1*06:02(0.16)	Non- toxin	0.5764
9	Probable outer membrane protein	Q9HYK0	405	419	AEARYAYLNAWLRLR	HLA-DRB5*01:01(0.01)	Non- toxin	0.7184
			84	98	ERDYRSYASTLSLEQ	HLA-DRB1*04:01(0.17)	Non- toxin	0.5761
10	Patatin-like protein, PlpD	Q9HYQ6	467	481	VGSRYFVAPFLFHEA	HLA-DPA1*01:03/DPB1*02:01(0.01)	Non- toxin	0.5079
			2	16	RRLLLVLLLLLPLSA	HLA-DPA1*03:01/DPB1*04:02(0.01)	Non- toxin	1.6032
			294	308	IDAGYRATTVLAARL	HLA-DQA1*01:02/DQB1*06:02(0.02)	Non- toxin	0.6612
			5	19	LLVLLLLLPLSALAA	HLA-DRB1*01:01(0.14)	Non- toxin	1.3696
			586	600	RQWDLRLNKALSFGA	HLA-DRB3*02:02(0.16)	Non- toxin	1.3838
11	Probable tonB-dependent receptor	Q9I473	369	383	QLSLRRDDNQQFGVH	HLA-DRB3*01:01(0.01)	Non- toxin	0.8807
			232	246	DNGLELDGTLLRAKS	HLA-DRB1*03:01(0.03)	Non- toxin	1.0111
			212	226	EPDRDGYRNLSGNLR	HLA-DRB5*01:01(0.14)	Non- toxin	0.7545

### Selection of B-cell epitopes in the OMPs for development of a chimeric vaccine

**Figure 3:**
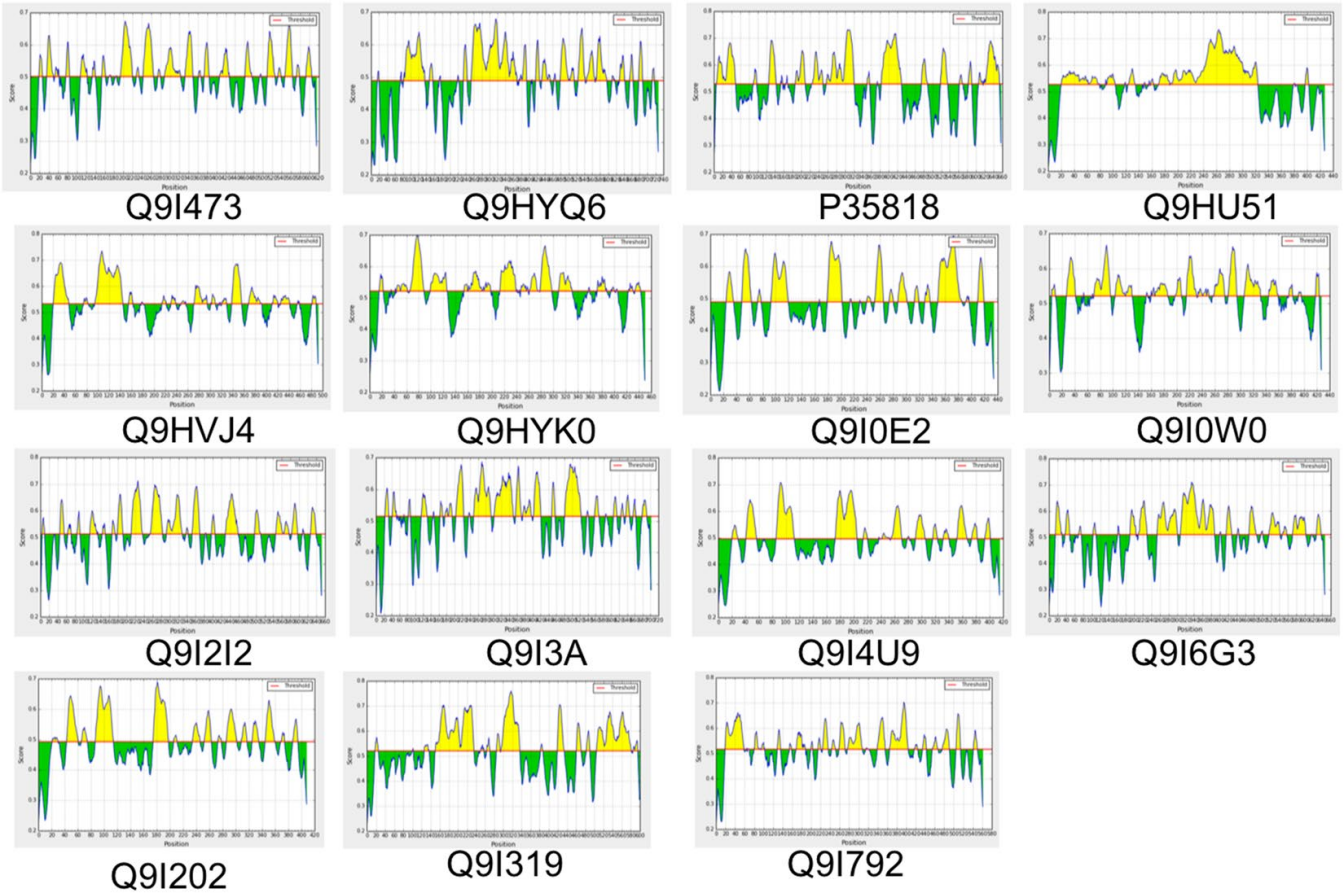
Identification of B cell epitopes of antigenic Proteins (UNIPORT ID) using Bepipred linear epitope server.

These 15 OMPs were analyzed to predict the B-cell epitope that enhances the humoral immune response. B-cell epitopes were identified by three different IEDB BepiPred linear epitope prediction servers, BCPREDS, and FBCPred. All 15 OMPs BepiPred linear diagrams have shown in Fig. 3. To improve the accuracy of epitope prediction, we selected epitopes which were predicted by all three tools. In total, 31 different epitopes (Table 4) of fifteen antigenic OMPs were selected from these three servers. These three servers gave the different length epitopes, and we have selected the epitopes on their antigenicity score by the vaxiJen server. In this, we selected 15 antigenic epitopes from all 15 proteins.

**Table 4:** Prediction of B cell epitopes using BCPRED, FBCPred and IEDB server and their antigenicity analysis.

S.No.	Protein ID	Start	BCPRED epitopes (score)	Antigenicity	Start	FBCPred epitopes (score)	Antigenicity	Start	IEDB server epitopes	Antigenicity
1	P35818	124	IVPNAEAKTEAGGGQSAPDR(0.998)	1.6543	124	IVPNAEAKTEAGGG(1)	2.0493	127	NAEAKTEAGGGQSAPDR	1.8726
2	Q9HU51	310	RVVARFGSQRGDDPRAKWDG(0.989)	0.9698	316	GSQRGDDPRAKWDG(1)	1.6403	190	LAATRKERQQALAKLNSDYRERDQKLKSRQQDQAELAKVLRTIEETLARQAREAAAAAERERQRALAAERERARQQQAAPGRVTSPPREPAPGPLVSSTGAVYGGAFGSARGKLPWPVNGRVVARFGSQRGDDPRA	0.6220
3	Q9I3A9	161	TRAPGESPGTRLKYTAGQRG(0.993)	1.4509	159	IITRAPGESPGTRL(0.989)	1.0269	164	PGESPGT	2.7224
		366	GLRPVCGRVDRNIRESRYDL(0.99)	1.3770	372	GRVDRNIRESRYDL(0.998)	1.1242	357, 375	QVLDQLRNSGLRPVCG, DRNIRESRYDLEMQDTLSLGDNLR	0.2116, 0.9067
4	Q9I6G3	219	NPGLNAEYPAGTGCCSDGES(0.998)	1.6557	218	TNPGLNAEYPAGTG(1)	1.2252	210	RELDIPLFTNPGLNAEYPAGT	0.8752
		374	EVSPDSGFDMPGNPESRRAG(0.996)	0.8327	379	SGFDMPGNPESRRA(0.999)	0.9380	309	RLVLEQTNADLLASTASGGALARQMEDQLQRQHQALTRLHDRRWLTLLGSNRPVGSFDGEVGAEGEVSPDSGFDMPGNPES	0.5776
5	Q9I202	90	DSGSGSGGTGLLPADGSAGG(0.998)	2.1982	90	DSGSGSGGTGLLPA(1)	2.4268	88	KLDSGSGSGGTGLLPADGSAGGSQDDYA	1.8319
6	Q9I4U9	257	RWARSTDEGGSRVNNRALNA(0.984)	1.6225	260	RSTDEGGSRVNNRA(1)	2.0610	256	IRWARSTDEGGSRVNN	1.6657
		323	DERSWQLRYDYDFAAIGLPG(0.941)	1.0355	324	ERSWQLRYDYDFA(1)	1.8151	333	YDFAAIGLP	0.0383
7	Q9I2I2	257	QRGRERRWRNSETGGPRSRY(0.999)	1.4075	256	HQRGRERRWRNSET(1)	1.1939	256	HQRGRERRWRNSETGGPRSRYYESRDVIE	0.9907
		439	SPEYAAVGGGGRFTIYGNPD(0.996)	0.8738	440	PEYAAVGGGGRFTI(1)	1.3966	430	YKAPSLKQLSPEYAAVGGGGRFTIYGNPDL	0.8609
		351	GEWRKEELEDRSVNTAGDAS(0.995)	1.2097	352	EWRKEELEDRSVNT(1)	1.0387	353	WRKEELEDRSVNTAGDAS	1.4536
		630	ADDDTHFTYAEPGRTFHLGL(0.737)	0.7266	631	DDDTHFTYAEPGRT(0.997)	0.5427	628	RLADDDTHFTYAE	−0.0919
8	Q9I319	310	RTTGQDSEEGGGAGNGAVGS(1)	2.9542	315	DSEEGGGAGNGAVG(1)	3.1690	296	SAGIRLGNNKSIQIRTTGQDSEEGGGAGNGAVGSLVDSRGLD	1.8233
		216	ANVVAVGDEPGKLRPGPQSS(0.997)	0.9639	222	GDEPGKLRPGPQSS(1)	1.1528	152	LELVEQTAQVLEQQYTLRSEKTGDLSVEIFPLRYAVAEDRKIEYRDDEIEAPGIASILSRVLSDANVVAVGDEPGKLRPGPQSSH	0.7862
		188	AEDRKIEYRDDEIEAPGIAS(0.964)	1.0594	188	AEDRKIEYRDDEIE(1)	1.3588		Similar to the above sequence	0.7862
9	Q9I792	50	QQLPGKGAPAAADASGGDER(1)	1.8121	48	ELQQLPGKGAPAAA(1)	0.8945	20	APFTSPGDRDLIRDRQQRLLDEQRKRLEELQQLPGKGAPA	0.1217
		390	GAQGRGHPQAGDPNARYDKY(1)	1.4353	392	QGRGHPQAGDPNAR(1)	1.8309	366	NHGRRIGSGFVNLDLGWQQGIGALGAQGRGHPQAGDPNAR	0.9329
10	Q9I0W0	281	IGSKYDQTARDGRGERVNLI(0.941)	2.1090	283	SKYDQTARDGRGER(0.969)	2.6857	282	GSKYDQTARDGRGE	2.3123
		77	IPNPDLSWSVEDTRQGNRQT(0.903)	1.4126	77	IPNPDLSWSVEDTR(0.9291)	0.9291	72	QQAGLIPNPDLSWSVEDTRQGNRQTS	1.0684
11	Q9HVJ4	339	SQIRDRYSEGGGDNSRSWDS(0.997)	1.8209	342	RDRYSEGGGDNSRS(1)	2.5326	339	SQIRDRYSEGGGDNSRSW	1.8534
		100	RATEDRTRTSNVSPTATLLG(0.997)	0.8936	101	ATEDRTRTSNVSPT(0.999)	1.4492	100	YRANRATEDRTRTSNVSPTATLLGEYGTRFSLAWVKQFRTADEAGRYRSD	0.7663
		245	VASQELNVEESTNQVDSARL(0.991)	1.1744	248	QELNVEESTNQVDS(1)	1.3340	249	ELNVEESTN	1.0580
		16	PVAASVNPALSPDVPSMARE(0.979)	0.6977	16	PVAASVNPALSPDV(1)	1.1248	22	NPALSPDVPSMAREQGRSVLLSEQVIDL	0.4140
12	Q9HYK0	164	FNQRAFEEGEGTRTDLLETR(0.997)	1.4105	169	FEEGEGTRTDLLET(1)	2.1021	164	RALETQLAFNQRAFEEGEGTRTDLLETRARLSLTRAEEIAASD	0.8831
		280	ASSSKTHSASESTYEQKYDT(0.997)	1.4625	285	THSASESTYEQKYD(1)	1.5251	280	ASSSKTHSASESTYEQKYD	1.5728
13	Q9I0E2	70	DLESAYTPGRVGFGLDLHGF(0.98)	1.1640	68	IVDLESAYTPGRVG(0.997)	0.8814	74	AYTPG	—
14	Q9HYQ6	90	ALSDAPPRKDVPFRRKQDDR(0.876)	0.8165	90	ALSDAPPRKDVPFR(0.943)	0.5882	83	LEMDWQQALSDAPPRKDVPFRRKQDDRDFLVKQKISFRDDGTLGLPLGVIQGQN	0.3697
15	Q9I473	168	YGTHQTLEGSAGVSGGAGNG(0.999)	2.1748	175	EGSAGVSGGAGNGW(1)	2.2144	170	THQTL	—
		201	INTKRAGTAGYEPDRDGYRN(0.998)	1.2803	207	GTAGYEPDRDGYRN(1)	1.3169	195	SFDTAGINTKRAGTAGYEPDRDGYRN	1.3263
		268	NLVGGRARFTPFDPWLVTLQ(0.791)	0.9480	271	GGRARFTPFDPWLV(0.976)	1.0753	275	RFTPFD	—

### Physiochemical analysis of epitopes

To explore the epitopes physiochemical properties, we have used GRAVY (grand average of hydropathy) analysis using ProtParam tool. Firstly, we have selected epitopes with the high antigenic score from each 15 OMPs. After shortlisting of high scored 40 (13 + 12 + 15) epitopes for MHC I, II and B cell (Table 5), we have proceeded for GRAVY analysis. In GRAVY result, we have gained the negative value score of total 28 (5 + 8 + 15) epitopes of MHC I, II and B cell respectively. These shortlisted hydrophilic epitopes may be present in the outer surface and have more chance to elicit the high immunogenicity in the host cell. These hydrophilic epitopes were selected to design a chimeric vaccine construct.

**Table 5:** Comparative prediction of MHC I, II and B cell epitopes and epitopes physiochemical analysis.

S.No.	Name	Start	MHC II epitope	Hydro-phobicity	Start	MHC I Epitope	Hydro-phobicity	Start	B cell epitope	Hydrophobicity
1	P35818,Type II secretion system protein D	74	GETFVVDPRVKGQVS	−0.213	49	QEAHWTINL	−0.578	124	IVPNAEAKTEAGGG	−0.271
2	Q9I3A9, Uncharacterized protein	665	ALELAGVLQQRLDDQ	−0.193	458	SPRLAVNYL	0.211	164	PGESPGT	−1.286
3	Q9I6G3, Uncharacterized protein	471	GGPVLLDQRLSGDTS	−0.300	542	LEASLGWRL	0.344	219	NPGLNAEYPAGTGCCSDGES	−0.675
4	Q9I202, Pyroglutatmate porin OpdO	266	RAFGALFSLRLGAHA	0.693	319	RSWQLRYDY	−1.833	90	DSGSGSGGTGLLPA	−0.057
5	Q9I4U9, Probable porin	58	QGFLLRYESGYTEGL	−0.407	325	RSWQLRYDY	−1.833	260	RSTDEGGSRVNNRA	−1.757
					64	YESGYTEGL	−0.900			
6	Q9I2I2, Probable TonB-dependent receptor	10	LPPLRLARAVPFLYL	0.967	522	LELGGGVDL	0.822	353	WRKEELEDRSVNTAGDAS	−1.439
7	Q9I792, Uncharacterized protein	519	GNAIELDARGRYFAA	−0.220	554	IYFRVDAFF	1.067	392	QGRGHPQAGDPNAR	−1.929
8	Q9HVJ4, Probable outer membrane protein	204	DAKYRAGAAALSDRL	−0.387	119	GEYGTRFSL	−0.556	342	RDRYSEGGGDNSRS	−2.314
9	Q9HYK0, Probable outer membrane protein	405	AEARYAYLNAWLRLR	−0.360	356	LAKVRAYEM	0.033	169	FEEGEGTRTDLLET	−1.036
10	Q9HYQ6, Patatin-like protein, PlpD	467	VGSRYFVAPFLFHEA	0.593	470	RYFVAPFLF	1.200	90	ALSDAPPRKDVPFRRKQDDR	−1.725
		2	RRLLLVLLLLLPLSA	1.920						
11	Q9I473, Probable tonB-dependent receptor	232	DNGLELDGTLLRAKS	−0.513	590	FGADHETAY	−0.689	175	EGSAGVSGGAGNGW	−0.293
12	Q9I0E2, OpdB proline porin	—	—		137	GEMTVETPVF	0.270	70	DLESAYTPGRVGFGLDLHGF	−0.060
13	Q9I0W0, Outer membrane protein CzcC	—	—		334	LLRLRSEAV	0.456	283	SKYDQTARDGRGER	−2.371
14	Q9HU51, Uncharacterized protein	—	—		—	—		316	GSQRGDDPRAKWDG	−2.114
15	Q9I319, Uncharacterized protein	—	—		—	—		315	DSEEGGGAGNGAVG	−0.671

### Selected epitopes were analyzed for MHC restriction and cluster analysis

**Figure 4:**
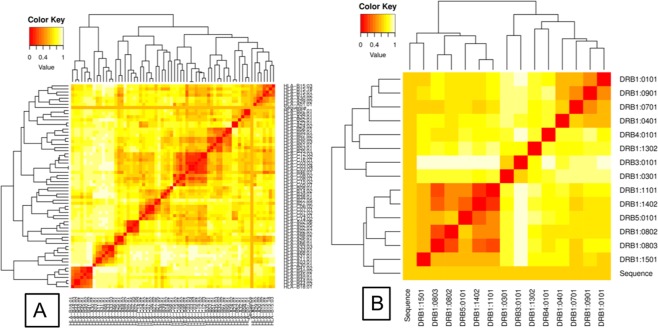
Cluster analysis of the HLA alleles for both MHC molecules through heat map representation. (**A**) Representing the cluster of the MHC-I (QEAHWTINL, RSWQLRYDY, YESGYTEGL, GEYGTRFSL, and FGADHETAY) epitopes. (**B**) Representing the cluster of MHC-II molecules (GETFVVDPRVKGQVS, ALELAGVLQQRLDDQ, GGPVLLDQRLSGDTS, QGFLLRYESGYTEGL, GNAIELDARGRYFAA, DAKYRAGAAALSDRL, AEARYAYLNAWLRLR, and DNGLELDGTLLRAKS) epitopes.

After physiochemical analysis, the selected epitopes were validated for the MHC interaction analysis using the MHCcluster. Interacted alleles were further assessed by cluster analysis and the result is shown as a heat map (Fig. 4) and dynamic tree (Supplementary Figure SF 2) concerning to MHC class I and MHC class II. The epitopes are clustered by interaction with HLA. Red color suggesting strong interaction while yellow color indicates weak interaction.

### Construction of chimeric vaccine

All shortlisted epitopes were used to design the chimeric vaccine. Using different analysis, we were shortlisted 5 different (QEAHWTINL, RSWQLRYDY, YESGYTEGL, GEYGTRFSL, and FGADHETAY) MHC I epitopes from 15 OMPs. Eight different MHC II epitopes (GETFVVDPRVKGQVS, ALELAGVLQQRLDDQ, GGPVLLDQRLSGDTS, QGFLLRYESGYTEGL, GNAIELDARGRYFAA, DAKYRAGAAALSDRL, AEARYAYLNAWLRLR, and DNGLELDGTLLRAKS) were selected using different analysis. From 15 OMPs, we have shortlisted 15 epitopes for B-cell (IVPNAEAKTEAGGG, PGESPGT, NPGLNAEYPAGTGCCSDGES, DSGSGSGGTGLLPA, RSTDEGGSRVNNRA, WRKEELEDRSVNTAGDAS, QGRGHPQAGDPNAR, RDRYSEGGGDNSRS, FEEGEGTRTDLLET, ALSDAPPRKDVPFRRKQDDR, EGSAGVSGGAGNGW, DLESAYTPGRVGFGLDLHGF, SKYDQTARDGRGER, GSQRGDDPRAKWDG, and DSEEGGGAGNGAVG). For vaccines construct, we have joined all epitopes with the help of linkers. To increase the vaccine immunogenicity, we have also added different adjuvants and PADRE sequence with the epitope sequences. We were made 4 different vaccines construct (VT1, VT2, VT3, and VT4). VT1 vaccine was merged with HBHA adjuvant, VT2 vaccine with HABA conserved sequence, VT3 vaccine with beta-defensin adjuvant and VT4 vaccine with 50 ribosomal L7/L12 protein. All detailed vaccine construct sequence was shown in Table 6.

**Table 6:** Characteristic properties of vaccine constructs determined using ProtParam server.

S.No.	Name	Sequence of vaccine	Mol wt (kDa)	PI	GRAVY	Aliphati c Index	Instab ility index	Negati ve amino acid	Positive amino acid
1	VT1	EAAAKMAENPNIDDLPAPLLAALGAADLALATVNDLIANLRERAEETRAETRTRVEERRARLTKFQEDLPEQFIELRDKFTTEELRKAAEGYLEAATNRYNELVERGEAALQRLRSQTAFEDASARAEGYVDQAVELTQEALGTVASQTRAVGERAAKLVGIELEAAAKAKFVAAWTLKAAAHEYGAEALERAGGETFVVDPRVKGQVSGGGSQEAHWTINLGGGSIVPNAEAKTEAGGGGGGSALELAGVLQQRLDDQGGGSPGESPGTGGGSGGPVLLDQRLSGDTSGGGSNPGLNAEYPAGTGCCSDGESGGGSRSWQLRYDYGGGSDSGSGSGGTGLLPAGGGSQGFLLRYESGYTEGLGGGSRSTDEGGSRVNNRAGGGSWRKEELEDRSVNTAGDASGGGSGNAIELDARGRYFAAGGGSQGRGHPQAGDPNARGGGSDAKYRAGAAALSDRLGGGSGEYGTRFSLGGGSRDRYSEGGGDSRSGGGSAEARYAYLNAWLRLRGGGSFEEGEGTRTDLLETGGGSALSDAPPRKDVPFRRKQDDRGGGSDNGLELDGTLLRAKSGGGSFGADHETAYGGGSEGSAGVSGGAGNGWGGGSDLESAYTPGRVGFGLDLHGFGGGSSKYDQTARDGRGERGGGSGSQRGDDPRAKWDGGGGSDSEEGGGAGNGAVGHEYGAEALERAGAKFVAAWTLKAAAHEYGAEALERAG	72.1(715 a.a chain)	4.88	−0.665	57.93	38.75	75	104
2	VT2	EAAAKMAENSNIDDIKAPLLAALGAADLALATVNELITNLRERAEETRRSRVEESRARLTKLQEDLPEQLTELREKFTAEELRKAAEGYLEAATSELVERGEAALERLRSQQSFEEVSARAEGYVDQAVELTQEALGTVASQVEGRAAKLVGIELEAAAKAKFVAAWTLKAAAHEYGAEALERAGGETFVVDPRVKGQVSGGGSQEAHWTINLGGGSIVPNAEAKTEAGGGGGGSALELAGVLQQRLDDQGGGSPGESPGTGGGSGGPVLLDQRLSGDTSGGGSNPGLNAEYPAGTGCCSDGESGGGSRSWQLRYDYGGGSDSGSGSGGTGLLPAGGGSQGFLLRYESGYTEGLGGGSRSTDEGGSRVNNRAGGGSWRKEELEDRSVNTAGDASGGGSGNAIELDARGRYFAAGGGSQGRGHPQAGDPNARGGGSDAKYRAGAAALSDRLGGGSGEYGTRFSLGGGSRDRYSEGGGDSRSGGGSAEARYAYLNAWLRLRGGGSFEEGEGTRTDLLETGGGSALSDAPPRKDVPFRRKQDDRGGGSDNGLELDGTLLRAKSGGGSFGADHETAYGGGSEGSAGVSGGAGNGWGGGSDLESAYTPGRVGFGLDLHGFGGGSSKYDQTARDGRGERGGGSGSQRGDDPRAKWDGGGGSDSEEGGGAGNGAVGHEYGAEALERAGAKFVAAWTLKAAAHEYGAEALERAG	71.0(706 a.a chain)	4.84	−0.653	59.07	41.00	73	104
3	VT3	EAAAKGIINTLQKYYCRVRGGRCAVLSCLPKEEQIGKCSTRGRKCCRRKKEAAAKAKFVAAWTLKAAAHEYGAEALERAGGETFVVDPRVKGQVSGGGSQEAHWTINLGGGSIVPNAEAKTEAGGGGGGSALELAGVLQQRLDDQGGGSPGESPGTGGGSGGPVLLDQRLSGDTSGGGSNPGLNAEYPAGTGCCSDGESGGGSRSWQLRYDYGGGSDSGSGSGGTGLLPAGGGSQGFLLRYESGYTEGLGGGSRSTDEGGSRVNNRAGGGSWRKEELEDRSVNTAGDASGGGSGNAIELDARGRYFAAGGGSQGRGHPQAGDPNARGGGSDAKYRAGAAALSDRLGGGSGEYGTRFSLGGGSRDRYSEGGGDSRSGGGSAEARYAYLNAWLRLRGGGSFEEGEGTRTDLLETGGGSALSDAPPRKDVPFRRKQDDRGGGSDNGLELDGTLLRAKSGGGSFGADHETAYGGGSEGSAGVSGGAGNGWGGGSDLESAYTPGRVGFGLDLHGFGGGSSKYDQTARDGRGERGGGSGSQRGDDPRAKWDGGGGSDSEEGGGAGNGAVGHEYGAEALERAGAKFVAAWTLKAAAHEYGAEALERAG	59.7(601 a.a chain	5.66	−0.712	49.85	37.82	67	75
4	VT4	EAAAKMAKLSTDELLDAFKEMTLLELSDFVKKFEETFEVTAAAPVAVAAAGAAPAGAAVEAAEEQSEFDVILEAAGDKKIGVIKVVREIVSGLGLKEAKDLVDGAPKPLLEKVAKEAADEAKAKLEAAGATVTVKEAAAKAKFVAAWTLKAAAHEYGAEALERAGGETFVVDPRVKGQVSGGGSQEAHWTINLGGGSIVPNAEAKTEAGGGGGGSALELAGVLQQRLDDQGGGSPGESPGTGGGSGGPVLLDQRLSGDTSGGGSNPGLNAEYPAGTGCCSDGESGGGSRSWQLRYDYGGGSDSGSGSGGTGLLPAGGGSQGFLLRYESGYTEGLGGGSRSTDEGGSRVNNRAGGGSWRKEELEDRSVNTAGDASGGGSGNAIELDARGRYFAAGGGSQGRGHPQAGDPNARGGGSDAKYRAGAAALSDRLGGGSGEYGTRFSLGGGSRDRYSEGGGDSRSGGGSAEARYAYLNAWLRLRGGGSFEEGEGTRTDLLETGGGSALSDAPPRKDVPFRRKQDDRGGGSDNGLELDGTLLRAKSGGGSFGADHETAYGGGSEGSAGVSGGAGNGWGGGSDLESAYTPGRVGFGLDLHGFGGGSSKYDQTARDGRGERGGGSGSQRGDDPRAKWDGGGGSDSEEGGGAGNGAVGHEYGAEALERAGAKFVAAWTLKAAAHEYG AEALERAG	68.0(686 a.a. chain)	4.86	−0.544	58.94	34.02	70	98

### Antigenicity, allergenicity, and solubility prediction of design vaccine constructs

Four vaccine constructs (VT1, VT2, VT3, and VT4) were further analyzed using the AlgPred, ANTIGENpro, VaxiJen 2.0 and SOLpro server (Supplementary Table ST-6). AlgPred server found all vaccine constructs have non-allergenic behavior. Antigenicity analysis of constructs was contained antigenicity score of more than 0.569 in ANTIGENpro and 1.5596 in VaxiJen 2.0 that suggests a good antigenic property of these four vaccines constructs. Using SOLpro, all four vaccine constructs were showed the good solubility (>0.9820) during its heterologous expression in the *E. coli*.

### Physicochemical analysis of design vaccine constructs

All four vaccine physicochemical properties were analyzed using ProtParam server. In this, all vaccine constructs molecular weight range have found between 59–72 kDa. Different physicochemical values of all vaccine construct demonstrate that the protein is stable in respective pH (Table 6). GRAVY (a hydropathic index) was found to be a negative value (−0.544), that suggest the hydrophilic nature of the design constructs and presents strong interactions with water molecules. The high aliphatic index range (49.85 to 59.07) of all vaccines construct indicates the protein stability in several temperatures. Three (VT1, VT3, and VT4) out of four vaccine constructs showed Instability score (<40) which explain that protein has good stability to initiate an immunogenic reaction. The total negative amino acid residues (Asp & Glu) and the total positive amino acid residues (Arg & Lys) were shown in the respective tables.

### Structure prediction of selected vaccine constructs

**Figure 5:**
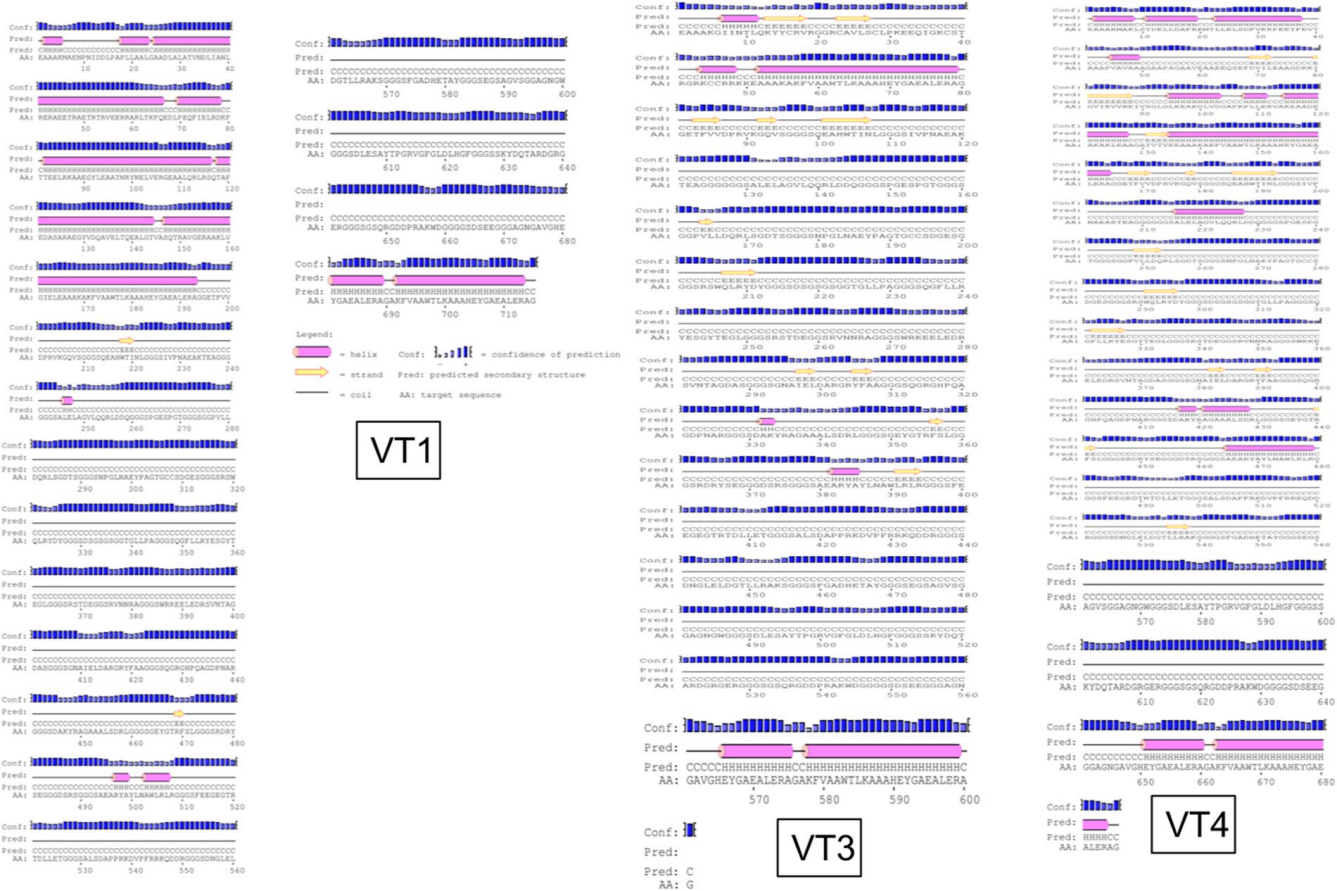
Secondary structure of vaccine constructs (VT1, VT3, and VT4) using PESIPRED server.

**Figure 6:**
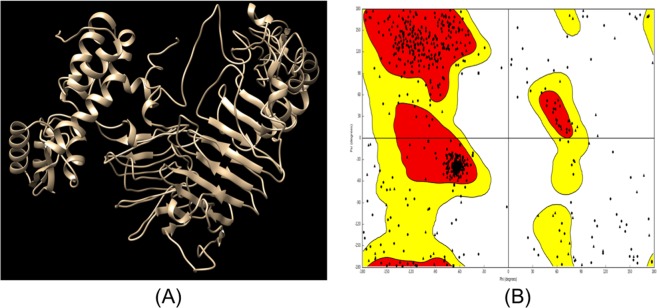
Tertiary structure prediction and validation of vaccine construct VT4. (**A**) The tertiary structure of model vaccine VT4. (**B**) Ramachandran plot of the modeled VT4 showing 92% residues in the allowed region.

Secondary structures of the final three vaccine constructs were predicted using PSIPRED. The secondary structure of VT1, VT3 and VT4 vaccine constructs are shown in Fig. 5. The structure of all vaccine constructs has alpha helix, beta-sheet, and beta turn. The model of VT1, VT3 & VT4 construct were generated and validated by the Ramachandran plot. The modeled structure and Ramachandran plot of VT4 is shown in Fig. 6.

### Interaction of vaccine constructs with HLA allele’s protein

A vaccine can interact with different HLA alleles of human populations. We have docked final 3 vaccine constructs (VT1, VT3, and VT4) with 10 different HLA allele’s proteins. VT4 have the least global binding energy value with different HLA alleles i.e. 1A6A (HLA-DR B1*03:01); −17.92, 1BX2 (HLA-DRB1*15:01); −22.69, 3C5J (HLA-DR B3*02:02); −14.16, 1H1S (HLA-DR B5*01:01); −5.26, 1SYS (HLA- B*44:03); −32.07, 1A1M (HLA-B*53:01); −20.03, 1XR8 (HLA-B*15:01); −11.61, 4O2E (HLA-B*39:01); −10.10, 5IM7 (HLA-B*58:01); −0.37, and 1ZSD (HLA-B*35:01); −10.10 as shown in (Supplementary Table ST-7). We have analyzed all three different constructs and finalized the one suitably, and best vaccine constructs i.e. VT4 that can be developed to control *P. aeruginosa* infection.

### Interaction and Molecular Dynamics Simulation of VT4 with TLR 4

**Figure 7:**
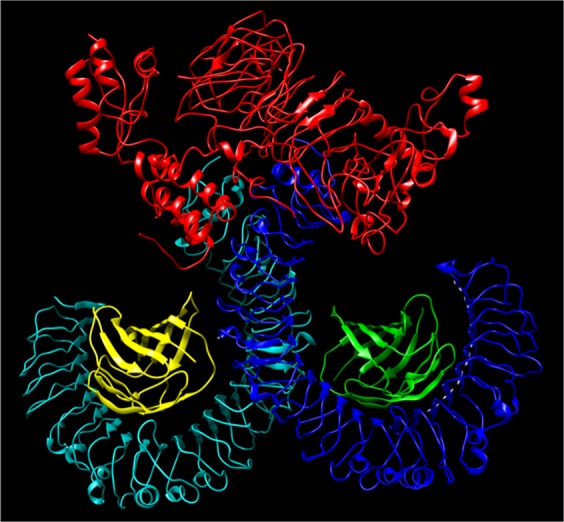
Docked complex of vaccine constructs VT4 with the human TLR4-MD2 complex. The vaccine construct VT-4 docked within the TLR-4 receptor.

**Figure 8:**
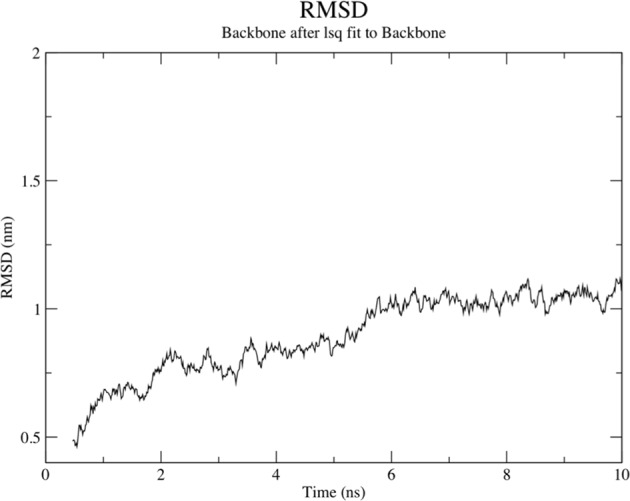
Molecular dynamics simulation of VT4- TLR4/MD2 complex. The result shows the RMSD obtained for the complex which showed that complex is stable at 6 ns.

The interaction between the TLR4 and adjuvants enhance the immune response. Hence, we have performed interaction study between VT4 with TLR 4/MD2 complex (PDB 3FXI). VT4 contains the adjuvant 50 s ribosomal L7/L12 protein. TLR 4 is agonist to an L7/L12 ribosomal protein that can increase the several immune responses in the host cell. The Patchdock docking result showed that negative (−11.11) binding energy that suggests a good interaction between VT4 and TLR-4/MD2 complex. This VT4-TLR4 complex further analyzed for docking using ClusPro. Best modeled ClusPro complex have minimum energy scored of −1009 which explained the interaction of VT4 with TLR-4 (Fig. 7). To further validate the interaction of the best-docked complex (from CluSPro), molecular dynamics simulation (MDS) was performed. MDS result also confirms that VT4-TLR-4/MD2 complex get stabilizes at 6 ns (Fig. 8) that further confirms the interaction between VT4 with TLR-4/MD2 complex.

### *In-silico* cloning of VT4 construct for its heterologous expression in *E. coli*

**Figure 9:**
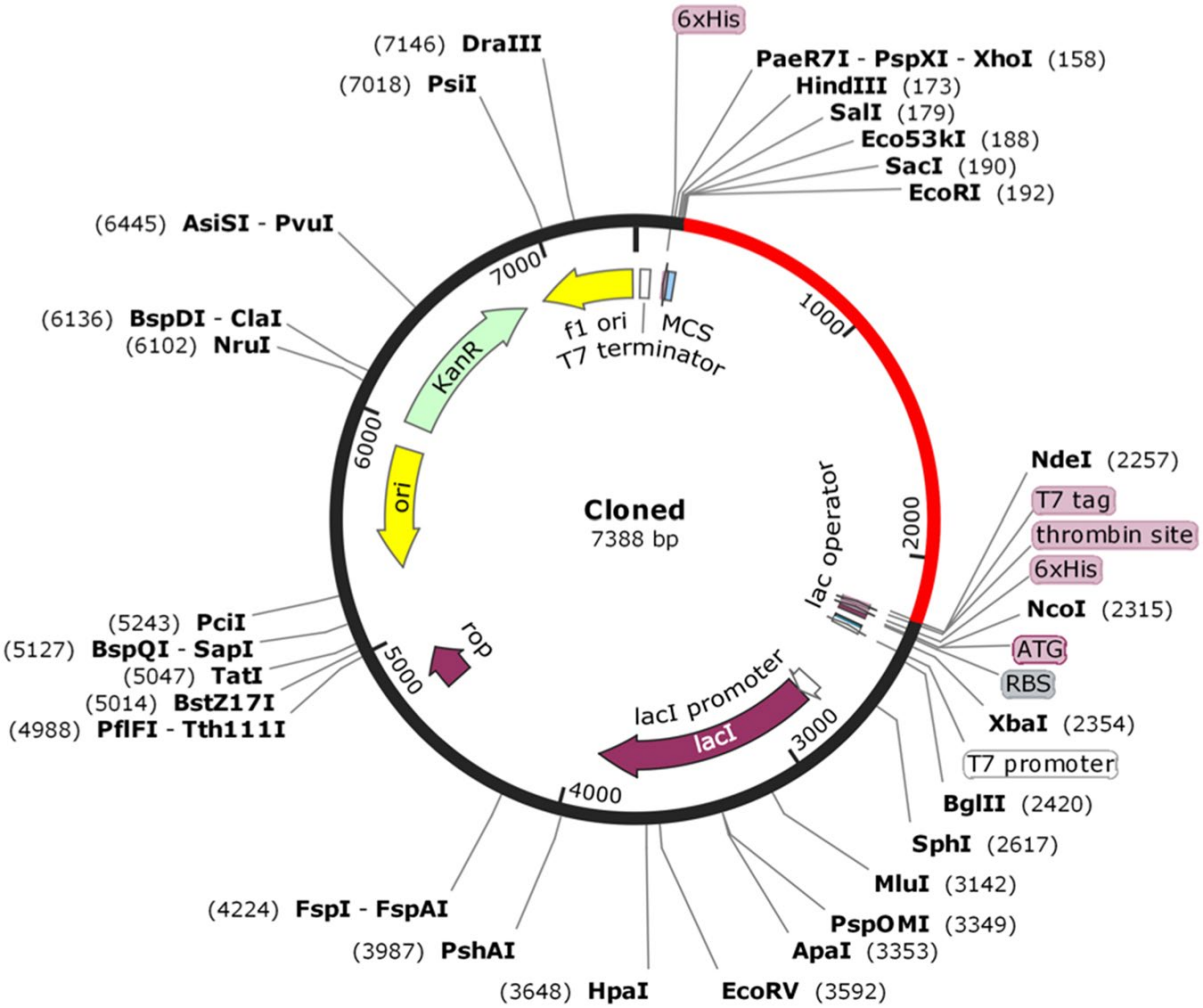
*In-silico* restriction cloning of gene sequence of final vaccine construct VT4 into pET28a expression vector showing VT4 sequence red colour surrounded between EcoRI (192) and NdeI (2257).

Chimeric vaccine cloning and expression within the expression vector were analyzed by Java Codon Adaptation Tool. Reverse translation generates cDNA sequence that will be used for *in-silico* cloning. Codon optimization analysis of VT4 construct showed 77.50% GC content of constructs. The CAI value indicates the heterologous expression of the selected gene. VT-4 have CAI value of 1.0 that suggests that it will be highly expressed in *E. coli* cell. The DNA sequence of restriction sites for EcoRI and NdeI restriction enzyme was added at 5′ and 3′ ends respectably. VT4 was *in-silico* cloned into pET28a vector for its heterologous expression in *E. coli* using EcoRI and NdeI restriction enzyme (Fig. 9).

## Discussion

The burden of disease caused by *P. aeruginosa* is the most common pathogen causing acute healthcare-associated pneumonia in the critically ill, elderly and immune-compromised person is substantial. Treatment is complicated by the organism’s resistance to multiple antibiotics and its capacity to form aggregates and biofilms on mucosal membranes and medical device surfaces. The establishment of persistent *P. aeruginosa* infection in those with chronic pulmonary disorders is also of considerable importance. Both acute and chronic *P. aeruginosa* infections are associated with significant morbidity, increased mortality and considerable cost to the health system and the community. There is no permanent cure and prevention for the multidrug-resistant *P. aeruginosa*. Studies have been done to screen herbal compounds^1,2^, nanomaterial^5,6^ and *in-silico* design therapeutics^7,8^ to find some alternative to the current therapeutics currently used against *P. aeruginosa*. Recently, proteome-based approaches (Subtractive genomics and reverse vaccinology) are used to design multi-epitope vaccine in *A. baumannii* by our group.

In the present work, we have shortlisted the non-redundant 20 strains with the reference proteome of *P. aeruginosa*. Using reference proteome i.e *P. aeruginosa* PAO1 strain, 5564 proteins were analyzed on the basis of different subtractive approaches. In the subtractive analysis, we have shortlisted the proteome on the basis of cellular localization, lipoproteins, a transmembrane helix, and classical and nonclassical secretary analysis. After the shortlisting, we have identified 15 antigenic outer membrane proteins. These 15 OMPs were classified on the basis of essentiality using DEG server, virulence factor using VFDB, and pathway-dependent and independent analysis using KEGG database. These 15 OMPs were selected for chimeric vaccine design using the reverse vaccinology. Antigenic, non-allergenic, nontoxic MHC I, MHC II and B cell epitopes were identified using different servers. All selected epitopes were merged with different adjuvants and linkers to enhance the immune responses. After joining with the different adjuvants, four vaccine constructs (VT1, VT2, VT3, and VT4) have been made. These vaccine constructs were further analyzed for their antigenic, allergenic and toxicity behavior. The non-toxic and non-allergenic vaccine may be the good immunotherapy against the pathogenic *P. aeruginosa*. On the basis of physiochemical behavior, we have shortlisted the three vaccine constructs (VT1, VT3, and VT4) for further analysis. Furthermore, docking based interaction analysis was performed to elucidate the binding affinity of designed vaccine with the different HLA molecules i.e. DRB1*03:01, DRB1*15:01, DRB3*02:02, DRB5*01:01, HLA-B*44:03, HLA-B*53:01, HLA-B*15:01, HLA-B*39:01, HLA-B*58:01 and HLA-B*35:01. With the help of docking analysis, we have shortlisted the VT4 as a potential vaccine that may enhance the immune response in the host cell. We have added different adjuvants to enhance immune response. We have also validated the interaction of VT4 with the TLR4/MD2 complex. Chimeric vaccine construct VT-4 against the *P. aeruginosa* may be used to generate the immune response and have the potential to interact with various HLA alleles. In the design vaccine constructs VT4, we have also added adjuvant L7/L12 ribosomal protein, Pan-DR epitopes, and linker along with multi-epitope sequences that may also enhance the significant *P. aeruginosa* specific immune responses. Therefore, in brief, we have taken possible significant factors that may induce the immunogenicity and feasibility of the vaccine construct VT-4.

## Conclusion

The availability of complete proteome of bacteria is facilitating many computational approaches. The data presented here demonstrate that stepwise prioritization of proteome using different comparative proteomics and reverse vaccinology approaches which are an effective way of rapidly reducing the unwanted number of *P. aeruginosa* proteins. This process is an efficient way of enriching the potential target, and for identifying those that are critical for normal cell function and have a virulent role in the host cell. Such a strategy will enable us to locate antigenic and immunogenic OMPs vaccine targets that have a role in pathogenesis. The antigenic, non-allergenic, nontoxic MHC I, MHC II and B cell epitopes were merged with different adjuvants and linkers to enhance the immune responses that further improves the effectiveness of the final chimeric multi-epitope vaccine VT-4. In addition to this, the designed vaccine VT-4 needs to be experimentally validated to ensure its efficacy against *P. aeruginosa* infections. The recommended vaccine (VT-4) need to be also validated in animal models before use against *P. aeruginosa*.

## Supplementary information

Supplementary Table and Figure

## Data Availability

All the data are available in the manuscript.
