# Supplementary material for: Prioritization of potential vaccine targets using comparative proteomics and designing of the chimeric multi-epitope vaccine against Pseudomonas aeruginosa
Source: Sci Rep. 2019 Mar 27;9:5240. doi: 10.1038/s41598-019-41496-4 (PMC6437148; doi:10.1038/s41598-019-41496-4)
Supplement: Supplementary file 1 — Supplementary Table and Figure [file 41598_2019_41496_MOESM1_ESM.pdf]

Supplementary Tables and Supplementary Figures

Title: Prioritization of potential vaccine targets using comparative proteomics and designing of the chimeric multi-epitope vaccine against *Pseudomonas aeruginosa*

Authors: Vandana Solanki, Monalisa Tiwari, and Vishvanath Tiwari*

Supplementary Table ST-1: Finalized shortlisted non-redundant strains of *P. aeruginosa*

S.No.	***P. aeruginosa* strains**	Uniport ID
1	*P. aeruginosa* ATCC 15692/PAO1 (Reference proteome)	UP000002438
2	*P. aeruginosa* stone 130	UP000017043
3	*P. aeruginosa* S742-C15-BS	UP000194726
4	*P. aeruginosa* MTB-1	UP000018564
5	*P. aeruginosa* S567-C10-BS	UP000194857
6	*P. aeruginosa* WH-SGI-V-07379	UP000054840
7	*P. aeruginosa* strain BL04	UP000017518
8	*P. aeruginosa* P28a	UP000216659
9	*P. aeruginosa* MH27	UP000018200
10	*P. aeruginosa* UCBPP-PA-14	UP000000653
11	*P. aeruginosa* WH-SGI-V-07064	UP000054218
12	*P. aeruginosa* F34365	UP000222703
13	*P. aeruginosa* VRFPA01	UP00017814
14	*P. aeruginosa* PA34	UP000223914
15	*P. aeruginosa* MH19	UP000043988
16	*P. aeruginosa* ATCC 9027	UP000222431
17	*P. aeruginosa* RNS-PA65	UP000222664
18	*P. aeruginosa* X9820	UP000226099
19	*P. aeruginosa* PA7	UP000001582
20	*P. aeruginosa* VRFPA03	UP000019105

Supplementary Table ST-2 Identification of *P. aeruginosa* unique pathways with respect to human host using KEGG database

S.No	Unique pathways of *P. aeruginosa*
1	01110 Biosynthesis of secondary metabolites
2	01120 Microbial metabolism in diverse environments
3	01130 Biosynthesis of antibiotics
4	01220 Degradation of aromatic compounds
5	00660 C5-Branched dibasic acid metabolism
6	00680 Methane metabolism
7	00300 Lysine biosynthesis
8	00460 Cyanoamino acid metabolism
9	00473 D-Alanine metabolism
10	00540 Lipopolysaccharide biosynthesis
11	00550 Peptidoglycan biosynthesis
12	00903 Limonene and pinene degradation
13	00281 Geraniol degradation
14	00523 Polyketide sugar unit biosynthesis
15	01053 Biosynthesis of siderophore group nonribosomal peptides
16	00332 Carbapenem biosynthesis
17	00261 Monobactam biosynthesis
18	00521 Streptomycin biosynthesis
19	00525 Acarbose and validamycin biosynthesis
20	00401 Novobiocin biosynthesis
21	00405 Phenazine biosynthesis
22	00362 Benzoate degradation
23	00627 Aminobenzoate degradation
24	00364 Fluorobenzoate degradation
25	00625 Chloroalkane and chloroalkene degradation
26	00361 Chlorocyclohexane and chlorobenzene degradation
27	00623 Toluene degradation
28	00622 Xylene degradation
29	00633 Nitrotoluene degradation
30	00643 Styrene degradation
31	00930 Caprolactam degradation
32	00626 Naphthalene degradation
33	02060 Phosphotransferase system (PTS)
34	03070 Bacterial secretion system
35	02020 Two-component system
36	02024 Quorum sensing
37	02025 Biofilm formation - Pseudomonas aeruginosa
38	02030 Bacterial chemotaxis

39	02040 Flagellar assembly
40	01501 beta-Lactam resistance
41	01502 Vancomycin resistance
42	01503 Cationic antimicrobial peptide (CAMP) resistance

Supplementary Table ST-3: Identification of MHC I epitopes using IEDB server, MHC-NP, NetCTLpan and NetMHCpan, and Class immunogenicity analysis

S.No.	Protein ID, name	start	end	Epitope	IEDB (MHC I binding)	MHC-NP	NetCTLpan	NetMHC	Class I immunogenicity
1	Q9HU51, Uncharacterized protein	105	114	LLAIQARAAY	HLA-B*15:01(0.15), HLA-B*35:01(0.2)	HLA-B*57:01 (0.8401)	-	HLA-B*15:01(0.3934)	0.17652
		181	190	GELDSRREAL	HLA-B*40:01(0.2)	HLA-B*57:01 (0.6271)	-	HLA-B*40:01(0.0545)	0.0641
		118	126	REEYLKLLL	HLA-B*40:01(0.2)	HLA-B*44:03 (0.3219)	HLA-B*40:01 (0.15)	HLA-B*40:01(0.0931)	-0.20086
		284	292	LVSSTGAVY	HLA-B*15:01(0.2)	HLA-B*53:01 (0.8156)	HLA-A*26:01 (0.80), HLA-B*15:01 (0.15), HLA-A*01:01 (0.30)	HLA-B*15:01(0.0467)	-0.09333
		139	148	TYYDYINKAR	HLA-A*33:01(0.2)	HLA-B*57:01 (0.4048)		HLA-A*24:02(0.4971)	-0.01811
		251	259	RQRALAAER	HLA-A*31:01(0.25)	HLA-B*44:03 (0.2556)			0.17372
2	Q9I6G3, Uncharacterized protein	193	201	VSNPALGAY	HLA-A*30:02(0.1)	HLA-B*53:01 (0.681)	HLA-A*01:01 (0.20), HLA-B*58:01(0.80), HLA-B*15:01(0.40)	HLA-A*26:01 (0.3064)	0.06586
		35	44	RLITDYPGRY	HLA-A*30:02(0.1)	HLA-B*57:01 (0.746)		HLA-A*03:01(0.4379)	0.14866
		236	244	GESFNGMDI	HLA-B*40:01(0.15)	HLA-B*53:01 (0.224)		HLA-B*40:01(0.2180)	-0.04554
		237	246	ESFNGMDIPV	HLA-A*68:02(0.15)	HLA-B*57:01 (0.5189)			0.02021
		635	643	NQAGYSLGV	HLA-A*02:06(0.2)	HLA-B*44:03 (0.229)			-0.10209
		561	569	RLQPYASLR	HLA-A*31:01(0.2)	HLA-B*53:01 (0.0727)	HLA-A*03:01(0.40)	HLA-A*03:01(0.1270)	-0.16163
		467	475	LQAAGGPVL	HLA-B*15:01(0.2)	HLA-B*53:01 (0.6489)	HLA-B*39:01(0.15), HLA-B*40:01(0.80)	HLA-B*39:01(0.0973)	0.13173
		66	74	QTSRQDFTW	HLA-B*58:01(0.2)	HLA-B*53:01 (0.7424)	HLA-B*58:01(0.05)	HLA-B*58:01(0.0076)	0.02794
		43	51	RYRGTASFA	HLA-A*30:01(0.2)	HLA-B*53:01 (0.172)			0.05431
		391	400	RAGVHLLGDY	HLA-A*30:02(0.2)	HLA-B*57:01 (0.9338)			0.10436
		505	514	RSGPLAGLDY	HLA-A*30:02(0.2)	HLA-B*57:01 (0.9042)			0.06264
		542	550	LEASLGWRL	HLA-B*40:01(0.25)	HLA-B*44:03 (0.2787)	HLA-B*40:01(0.10)	HLA-B*40:01(0.0556)	0.08451
		521	529	DFREDEALR	HLA-A*33:01(0.25)	HLA-B*53:01 (0.0711)			0.25994
		466	474	YLQAAGGPV	HLA-A*02:03(0.25)	HLA-B*53:01 (0.0566)	HLA-A*02:01(0.80)		0.09389
		66	74	QTSRQDFTW	HLA-B*57:01(0.25)	HLA-B*53:01 (0.7424)	HLA-B*58:01(0.05)	HLA-B*58:01(0.0076)	0.02794
		344	352	LTRLHDRRW	HLA-B*57:01(0.25)	HLA-B*57:01 (0.9626)		HLA-B*58:01(0.1614)	0.13194
3	Q9HVJ4, Probable outer membrane protein	134	142	RTADEAGRY	HLA-A*30:02(0.1)	HLA-B*44:03 (0.6287)	HLA-A*01:01(0.05), HLA-A*26:01(0.30)	HLA-A*01:01(0.0704)	0.22819
		188	197	QTISQVIGAY	HLA-A*30:02(0.1)	HLA-B*44:03 (0.7106))		HLA-A*26:01(0.0225)	-0.01913
		119	127	GEYGTRFSL	HLA-B*40:01(0.15)	HLA-B*44:03 (0.2256)	HLA-B*39:01(0.40), HLA-B*40:01(0.01)	HLA-B*39:01(0.1811)	0.12156
		164	172	TTAPLRLAR	HLA-A*68:01(0.2)	HLA-B*44:03 (0.4345)			0.05296
		190	198	ISQVIGAYR	HLA-A*31:01(0.2)	HLA-B*44:03 (0.3639)			0.1963
		306	315	EYLQRLIGSR	HLA-A*33:01(0.2)	HLA-B*44:03 (0.2162)			-0.02334
		85	93	AFNPKLVVR	HLA-A*31:01(0.25)	HLA-B*53:01 (0.1518)			-0.17474
4	Q9I0E2, OpdB proline porin	173	181	IQAGRFTAF	HLA-B*15:01(0.1)	HLA-B*53:01 (0.7975)	HLA-B*08:01(0.80), HLA-B*15:01(0.05)	HLA-B*15:01(0.0070), HLA-B*08:01(0.3041)	0.26302

		389	398	RYLFASGPFK	HLA-A*03:01(0.1), HLA-A*03:01(0.15)	HLA-B*57:01 (0.2756)		HLA-A*03:01 (0.0294)	0.08214
		33	42	DYLEWRLLNR	HLA-A*33:01(0.15)	HLA-B*57:01 (0.2147)			0.33967
		38	47	RLLNRNYFLY	HLA-A*30:02(0.15)	HLA-B*57:01 (0.7723)			0.12283
		223	231	FAAQLDNVW	HLA-B*53:01(0.2), HLA-B*58:01(0.2)	HLA-B*53:01 (0.4756)	HLA-B*58:01(0.40)	HLA-B*58:01(0.0397)	-0.07512
		3	12	RVRPVGRLAL	HLA-B*07:02(0.2), HLA-A*30:01(0.2)	HLA-B*57:01 (0.9738)			0.14106
		43	52	NYFLYNDYRR	HLA-A*33:01(0.2)	HLA-B*57:01 (0.1376)			0.06494
		340	349	SFMARYVSGR	HLA-A*33:01(0.2), HLA-A*31:01(0.25)	HLA-B*57:01 (0.5972)			-0.05179
		137	146	GEMTVETPVF	HLA-B*44:02(0.25)	HLA-B*57:01 (0.7617)	HLA-B*40:01(0.20)	HLA-B*40:01(0.1780)	0.17106
		267	275	TLAYSLQLK	HLA-A*03:01(0.25)	HLA-B*53:01 (0.2239)	HLA-A*03:01(0.80)	HLA-A*03:01(0.1285)	-0.2668
		126	135	KLRLGESLLK	HLA-A*03:01(0.25)	HLA-B*57:01 (0.9662)		HLA-B*27:05(0.4886)	-0.03543
5	Q9HYK0, Probable outer membrane protein	207	215	LEAMLGQAL	HLA-B*40:01(0.15)	HLA-B*44:03 (0.3383)	HLA-B*39:01 (0.80), HLA-B*40:01(0.15)	HLA-B*40:01(0.0379)	-0.2178
		259	267	EAAAYEVER	HLA-A*68:01(0.15)	HLA-B*53:01 (0.4454)			0.23606
		409	417	YAYLNAWLR	HLA-A*68:01(0.15)	HLA-B*53:01 (0.0795)			0.19863
		1	9	MNRLRACLL	HLA-B*08:01(0.2)		HLA-B*08:01(0.10)		0.04089
		356	364	LAKVRAYEM	HLA-B*08:01(0.2)	HLA-B*53:01 (0.3206)	HLA-B*08:01(0.30)	HLA-B*08:01(0.0303)	0.11415
		415	423	WLRLRQLAG	HLA-B*08:01(0.2)	HLA-B*53:01 (0.0234)			-0.0395
		23	32	GLLDAYQLAV	HLA-A*02:01(0.2)	HLA-B*07:02 (0.0096)		HLA-A*02:01(0.0732)	-0.04232
		199	207	RAAAARRTL	HLA-B*07:02(0.2)	HLA-B*57:01 (0.7068)	HLA-B*07:02(0.20)	HLA-B*07:02 (0.0826)	0.20525
		222	230	APIERFPAL	HLA-B*07:02(0.2)	HLA-B*07:02 (0.5309)	HLA-B*07:02(0.05), HLA-B*08:01(0.05), HLA-B*39:01(0.30)	HLA-B*07:02(0.0083)	0.31805
		275	284	RLSLYASSSK	HLA-A*03:01(0.2)	HLA-B*57:01 (0.8215)		HLA-A*03:01(0.1435)	-0.42237
		130	138	LAVRLFAAY	HLA-B*35:01(0.2)	HLA-B*44:03 (0.8667)	HLA-B*15:01(0.30)		0.22076
		410	419	AYLNAWLRLR	HLA-A*31:01(0.25)	HLA-B*57:01 (0.675)			0.27045
		330	338	AQAELDAQV	HLA-A*02:06(0.25)	HLA-B*44:03 (0.7056)			0.08887
6	Q9HYQ6, Patatin-like protein, PlpD	105	113	KQDDRDFLV	HLA-A*02:06(0.1)	HLA-A*02:01 (0.2008)		HLA-A*02:01(0.4343)	0.19312
		425	433	STFNLGGSY	HLA-A*30:02(0.1), HLA-A*68:01(0.15)	HLA-B*44:03 (0.8235)	HLA-B*15:01(0.30)	HLA-A*03:01(0.3294)	-0.01547
		470	478	RYFVAPFLF	HLA-A*23:01(0.1), HLA-A*24:02(0.1)	HLA-B*53:01 (0.4128)	HLA-A*24:02(0.01)	HLA-A*24:02(0.2738)	0.19952
		189	198	MSIPAVFAPV	HLA-A*68:02(0.1), HLA-A*02:06(0.15)	HLA-B*57:01 (0.5672)			0.24708
		60	69	SMGAVVGGLY	HLA-A*30:02(0.1), HLA-A*30:02(0.2)	HLA-B*44:03 (0.7914)			0.18479
		616	625	AEVVTSSFTL	HLA-B*40:01(0.15)	HLA-B*57:01 (0.6347)		HLA-B*40:01(0.1283)	-0.10261
		74	83	TPAELERIAL	HLA-B*07:02(0.15)	HLA-B*57:01 (0.8672)		HLA-B*07:02(0.0362)	0.38405
		313	322	KPKDLNSEAL	HLA-B*07:02(0.15)	HLA-B*57:01 (0.893)		HLA-B*07:02(0.0277)	-0.11315
		645	653	SLGRIVYYR	HLA-A*31:01(0.15), HLA-A*68:01(0.15)	HLA-B*44:03 (0.3347)		HLA-A*03:01(0.3680)	0.22626
		651	660	YYRRLTERSF	HLA-A*24:02(0.15)	HLA-B*57:01 (0.7592)		HLA-A*24:02(0.4874)	0.13715
		660	669	FLPLDFPLYL	HLA-A*02:01(0.2)	HLA-B*57:01 (0.5819)		HLA-A*02:01(0.0381)	0.09888
		694	703	SLMIGFDTPL	HLA-A*02:01(0.2)	HLA-B*53:01 (0.3825)			0.27108
		545	553	GYYELKYSF	HLA-A*23:01(0.2)	HLA-B*44:03 (0.5956)	HLA-A*24:02(0.10)	HLA-A*24:02(0.0170)	-0.21403
		476	484	FLFHEAQNV	HLA-A*02:01(0.2)	HLA-A*02:01	HLA-A*02:01(0.10)	HLA-A*02:01(0.0126)	0.10334

					(0.2671)			
		67	76	GLYASGYTPA	HLA-A*02:03(0.2)	HLA-B*57:01 (0.2829)			-0.06713
		603	611	WVFGGGYGR	HLA-A*68:01(0.2)	HLA-B*53:01 (0.113)			0.15368
		181	189	LPQAIRASM	HLA-B*07:02(0.2)	HLA-B*07:02 (0.3453)	HLA-B*08:01(0.80), HLA-B*07:02(0.05)	HLA-B*07:02(0.0078)	0.11645
		379	387	DYFDQVQYR	HLA-A*33:01(0.2)	HLA-B*53:01 (0.1365)			-0.11354
		720	728	FYLNLGQNF	HLA-A*23:01(0.25)	HLA-B*53:01 (0.6899)	HLA-A*24:02(0.10)	HLA-A*24:02(0.0138)	-0.09055
		440	449	RLGAEWLTR V	HLA-A*02:03(0.25)	HLA-B*57:01 (0.6941)		HLA-A*02:01(0.3154)	0.41613
		186	195	RASMSIPAVF	HLA-B*15:01(0.25)	HLA-B*57:01 (0.7732)		HLA-B*58:01(0.1804)	-0.2152
		476	484	FLFHEAQNV	HLA-A*02:03(0.25)	HLA-A*02:01 (0.2671)	HLA-A*02:01(0.10)	HLA-A*02:01(0.0126)	0.10334
		650	658	VYYRRLTER	HLA-A*31:01(0.25)	HLA-B*53:01 (0.0909)			0.1821
7	Q9I0W0, Outer membrane protein CzcC	392	401	RTLVGVRAQY	HLA-A*30:02(0.1)	HLA-B*57:01 (0.9746)		HLA-B*58:01(0.3341)	0.1252
		233	242	VPASITRGAL	HLA-B*07:02(0.15)	HLA-B*57:01 (0.8319)		HLA-B*07:02(0.0245)	0.11381
		152	160	RVRLAKTSL	HLA-B*07:02(0.2)	HLA-B*57:01 (0.2006)	HLA-B*07:02(0.10), HLA-B*08:01(0.80)	HLA-B*07:02(0.0692)	-0.22316
		258	266	EVARGEAQV	HLA-A*68:02(0.2)	HLA-B*53:01 (0.4954)			0.15737
		373	381	MTRGFEMGK	HLA-A*30:01(0.2)	HLA-B*53:01 (0.1676)		HLA-A*03:01(0.1147)	0.13075
		273	281	RIPNLTVSI	HLA-A*32:01(0.2)	HLA-A*02:01 (0.2798)			-0.04619
		334	342	LLRLRSEAV	HLA-B*08:01(0.2)	HLA-A*02:01 (0.0807)	HLA-B*08:01(0.10)	HLA-B*08:01(0.0564)	0.00767
		294	302	GERVNLIGL	HLA-B*40:01(0.25)	HLA-B*53:01 (0.0519)	HLA-B*40:01(0.40)	HLA-B*40:01(0.1368)	0.17372
8	Q9I2I2, Probable TonB-dependent receptor	380	388	DEIAFGPDW	HLA-B*44:02(0.1), HLA-B*44:03(0.1)	HLA-B*53:01 (0.6272)			0.23207
		14	23	RLARAVPFLY	HLA-A*30:02(0.1)	HLA-B*57:01 (0.8863)	HLA-A*02:01(0.30)	HLA-A*03:01(0.3228)	0.22496
		522	530	LELGGGVDL	HLA-B*40:01(0.15)	HLA-B*53:01 (0.5935)	HLA-B*40:01(0.10)	HLA-B*40:01(0.0361)	0.1432
		68	77	RPVRDLQEAL	HLA-B*07:02(0.15)	HLA-B*57:01 (0.9091)	HLA-B*07:02(0.80)	HLA-B*07:02(0.0263)	0.0746
		407	416	SPRLYLLHHL	HLA-B*07:02(0.15)	HLA-B*57:01 (0.9066)		HLA-B*07:02(0.3545)	0.027
		411	419	YLLHHLSDV	HLA-A*02:03(0.15)	HLA-A*02:01 (0.5492)	HLA-A*02:01(0.80)	HLA-A*02:01(0.0792)	-0.07665
		304	312	RTYRNRLER	HLA-A*31:01(0.15)	HLA-B*57:01 (0.1241)	HLA-A*03:01(0.30)	HLA-A*03:01(0.1019)	0.14244
		400	409	EAFGWESSPR	HLA-A*68:01(0.15)	HLA-B*57:01 (0.7222)			0.08347
		451	460	FTIYGNPDLK	HLA-A*68:01(0.15)	HLA-B*57:01 (0.7442)			0.06798
		287	296	RWSLAHNGQ W	HLA-B*58:01(0.15)	HLA-B*57:01 (0.7864)		HLA-B*58:01(0.1762)	-0.04043
		162	170	ITRRATDTW	HLA-B*57:01(0.15)	HLA-B*53:01 (0.6409)	HLA-B*58:01(0.05)	HLA-B*58:01(0.0158)	0.18492
		21	30	FLYLLPCLAL	HLA-A*02:01(0.2)	HLA-B*57:01 (0.2243)			-0.07121
		404	412	WESSPRLYL	HLA-B*40:01(0.2)	HLA-B*53:01 (0.191)	HLA-B*40:01(0.10)	HLA-B*39:01(0.3844)	0.19377
		23	31	YLLPCLALA	HLA-A*02:01(0.2), HLA-A*02:06(0.25)	HLA-B*53:01 (0.1066)	HLA-A*02:01(0.20)	HLA-A*02:01(0.3338)	-0.05116
		201	209	VPGKLGLAL	HLA-B*07:02(0.2)	HLA-B*07:02 (0.1291)	HLA-B*07:02(0.15)	HLA-B*07:02(0.0165)	-0.1714
		595	603	YALPAYSLW	HLA-B*58:01(0.2), HLA-B*57:01(0.25)	HLA-B*53:01 (0.8414)	HLA-A*24:02(0.40)	HLA-B*58:01(0.0054)	0.12624
		436	445	KQLSPEYAAV	HLA-A*02:06(0.25)	HLA-B*57:01 (0.7767)	HLA-A*02:01(0.80)		-0.03097
		574	583	FSAQLRTEYV	HLA-A*68:02(0.25)	HLA-B*57:01 (0.9515)	HLA-A*26:01(0.30)		0.05462
		364	372	NTAGDASAR	HLA-A*68:02(0.25)	HLA-B*53:01			-0.01153

						(0.1965)			
		49	57	KLRDAPASV	HLA-A*02:03(0.25)	HLA-A*02:01 (0.7401)	HLA-A*02:01(0.80)	HLA-A*02:01(0.2252)	0.00314
		288	296	WSLAHNGQW	HLA-B*57:01(0.25)	HLA-B*53:01 (0.798)	HLA-B*58:01(0.20)	HLA-B*58:01(0.0741)	0.0221
		428	436	RGYKAPSLK	HLA-A*03:01(0.25)	HLA-B*53:01 (0.1262)	HLA-A*03:01(0.30)	HLA-A*03:01(0.1234)	-0.33664
9	Q9I4U9, Probable porin	293	302	RMSGDSGFAY	HLA-A*30:02(0.1)	HLA-B*53:01 (0.52)		HLA-A*03:01(0.4685)	-0.00554
		325	333	RSWQLRYDY	HLA-A*30:02(0.15)	HLA-B*44:03 (0.598)	HLA-B*58:01(0.40)	HLA-B*58:01(0.2830)	0.0031
		302	311	YLAGTDPYLV	HLA-A*02:01(0.15), HLA-A*02:03(0.15), HLA-A*02:06(0.25)	HLA-B*57:01 (0.5534)		HLA-A*02:01(0.0559)	0.08616
		64	72	YESGYTEGL	HLA-B*40:01(0.15)	HLA-B*53:01 (0.4653)	HLA-B*39:01(0.80)	HLA-B*39:01(0.2399)	0.11764
		6	15	VPMLAAGLAL	HLA-B*07:02(0.15), HLA-A*02:03(0.2)	HLA-B*57:01 (0.3859)		HLA-B*07:02(0.0313)	0.05344
		285	293	HAFGLGYQR	HLA-A*68:01(0.15)	HLA-B*44:03 (0.53)			0.0224
		343	351	LTFMSRYLR	HLA-A*68:01(0.15), HLA-A*31:01(0.15)	HLA-B*44:03 (0.083)			-0.26068
		273	281	ALNALFTYR	HLA-A*31:01(0.15)	HLA-B*44:03 (0.7219)	HLA-A*03:01(0.40)	HLA-A*03:01(0.1388)	0.16727
		319	327	FANKDERSW	HLA-B*53:01(0.2)	HLA-B*53:01 (0.9372)	HLA-B*58:01(0.80)	HLA-B*58:01(0.0270)	-0.15623
		33	41	ELRNFYFNR	HLA-A*33:01(0.2)	HLA-B*44:03 (0.2008)			0.21583
		132	140	TLMPRLPVV	HLA-A*02:01(0.2)	HLA-A*02:01 (0.6455)	HLA-A*02:01(0.10)	HLA-B*08:01(0.0773) HLA-A*02:01(0.0137)	-0.01344
		226	234	YANLEDFYR	HLA-A*68:01(0.2), HLA-A*33:01(0.2)	HLA-B*53:01 (0.3909)			0.20176
		206	215	RFDFAGGTYR	HLA-A*31:01(0.2)	HLA-B*53:01 (0.6491)	HLA-A*01:01(0.80)		0.2586
		44	52	RQPGASQSY	HLA-B*15:01(0.2)	HLA-B*44:03 (0.3557)	HLA-B*15:01(0.40)	HLA-B*15:01(0.0115)	-0.28155
		134	142	MPRLPVVQF	HLA-B*07:02(0.2)	HLA-B*35:01 (0.8115)	HLA-B*07:02(0.05)	HLA-B*07:02(0.0156)	0.00086
		48	56	ASQSYSEEW	HLA-B*58:01(0.2)	HLA-B*53:01 (0.8914)	HLA-B*58:01(0.15)	HLA-B*58:01(0.0256)	-0.2204
		250	258	QSLKSDIRW	HLA-B*58:01(0.2)	HLA-B*57:01 (0.7409)	HLA-B*58:01(0.10)	HLA-B*58:01(0.0073)	-0.21826
		120	129	KLRVSHSTLK	HLA-A*03:01(0.2)	HLA-B*57:01 (0.9575)		HLA-A*03:01(0.0749)	-0.20071
		308	316	PYLVNFVQI	HLA-A*23:01(0.25)	HLA-B*53:01 (0.115)	HLA-A*24:02 (0.15)	HLA-A*24:02(0.1399)	0.109
		36	44	NFYFNRDYR	HLA-A*33:01(0.25)	HLA-B*44:03 (0.0702)			0.17558
		55	63	EWAQGFLLR	HLA-A*33:01(0.25)	HLA-B*44:03 (0.4547)			0.0235
10	Q9I319, Type III secretion outer membrane protein PscC	470	478	VPWLGDIPY	HLA-B*35:01(0.1)	HLA-B*35:01 (0.2663)			0.22046
		81	89	ASLYNLGWY	HLA-A*30:02(0.15)	HLA-B*44:03 (0.3651)			0.13396
		89	98	YYDGTVLYVF	HLA-A*23:01(0.15), HLA-A*24:02(0.15)	HLA-B*53:01 (0.6136)		HLA-A*24:02(0.0578)	0.12986
		32	40	YVAQGESLR	HLA-A*68:01(0.15)	HLA-B*53:01 (0.4613)			-0.12271
		123	131	TAAGIWEAR	HLA-A*68:01(0.15)	HLA-B*53:01 (0.5676)			0.49227
		120	128	RALTAAGIW	HLA-B*57:01(0.15)	HLA-B*53:01 (0.7294)	HLA-B*58:01(0.20)	HLA-B*58:01(0.0267)	0.21675
		200	209	IEAPGIASIL	HLA-B*40:01(0.2)	HLA-B*57:01 (0.9009)	HLA-B*40:01(0.30)	HLA-B*40:01(0.0835)	0.13911
		338	346	FLLAKVTLL	HLA-A*02:01(0.2)	HLA-A*02:01 (0.5177)	HLA-A*02:01(0.05)	HLA-A*02:01(0.0174)	-0.10909
		179	187	EIFPLRYAV	HLA-A*68:02(0.2)	HLA-B*44:03 (0.4313)			0.0845
		80	88	MASLYNLGW	HLA-B*57:01(0.2), HLA-B*57:01(0.2)	HLA-B*53:01 (0.4774)	HLA-B*58:01(0.05)	HLA-B*58:01(0.0339)	-0.06411
11	Q9I473, Probable tonB-dependent	521	529	LPRRARRMF	HLA-B*07:02(0.1)	HLA-B*57:01 (0.407)	HLA-B*08:01(0.80)	HLA-B*07:02(0.1439)	0.09678

	receptor								
		572	580	SEYRLNDEW	HLA-B*44:03(0.1)	HLA-B*44:03 (0.442)			0.11121
		323	332	AEGHVLTLGY	HLA-B*44:03(0.15)	HLA-B*57:01 (0.8767)			0.11993
		93	101	TESDHVLVL	HLA-B*40:01(0.15)	HLA-B*53:01 (0.6523)	HLA-B*39:01(0.80)	HLA-B*39:01(0.1398)	0.05374
		197	205	DTAGINTKR	HLA-A*68:01(0.15)	HLA-B*53:01 (0.1578)			0.07707
		401	409	TVSYGTAFK	HLA-A*68:01(0.15), HLA-A*11:01(0.2)	HLA-B*53:01 (0.2427)	HLA-B*58:01(0.80)	HLA-A*03:01(0.0948)	0.11354
		302	311	RFYSRFDTRR	HLA-A*31:01(0.15)	HLA-B*57:01 (0.6709)			0.08061
		350	359	RLNKGWFAQ Y	HLA-A*30:02(0.15)	HLA-B*57:01 (0.7329)		HLA-B*15:01(0.1969)	0.10514
		486	494	VELVLGSQW	HLA-B*44:03(0.15)	HLA-B*53:01 (0.7497)			-0.14826
		392	400	YALSDALRF	HLA-B*53:01(0.2), HLA-A*02:01(0.25)	HLA-B*53:01 (0.8277)	HLA-B*58:01(0.80)	HLA-B*58:01(0.0957)	-0.09076
		297	306	AYQDGRFYSR	HLA-A*31:01(0.2)	HLA-B*44:03 (0.4147)			0.07854
		130	138	VVRGPRSSL	HLA-B*07:02(0.2)	HLA-B*07:02 (0.6918)	HLA-B*07:02(0.20)	HLA-B*07:02(0.0376) HLA-B*08:01(0.3152)	-0.14746
		590	598	FGADHETAY	HLA-B*35:01(0.2)	HLA-B*53:01 (0.9534)	HLA-A*26:01(0.80)	HLA-B*15:01(0.2933)	0.21639
		608	616	YLSVRYQAL	HLA-B*08:01(0.2)	HLA-B*53:01 (0.1455)	HLA-B*08:01(0.01)	HLA-B*08:01(0.0090)	-0.04014
12	Q9I792, Uncharacterized protein	481	489	VEWAPLRPW	HLA-B*44:02(0.1), HLA-B*44:03(0.1)	HLA-B*44:03 (0.5076)			0.12723
		158	166	SPRELTMSF	HLA-B*07:02(0.1)	HLA-B*44:03 (0.3969)	HLA-B*07:02 (0.01)	HLA-B*07:02(0.0060)	-0.10157
		269	277	HSDSQSLFY	HLA-A*01:01(0.1)	HLA-B*53:01 (0.7565)	HLA-A*01:01(0.01)		-0.36876
		491	499	QEYGVAFAY	HLA-B*44:03(0.1)	HLA-B*44:03 (0.7629)	HLA-B*40:01(0.80)		0.2 3159
		76	84	IELEGAGHL	HLA-B*40:01(0.15)	HLA-B*44:03 (0.6337)	HLA-B*40:01(0.05)		0.21448
		234	242	GEQQMGLGL	HLA-B*40:01(0.15)	HLA-B*44:03 (0.2411)	HLA-B*40:01(0.15)		-0.8282
		554	562	IYFRVDAFF	HLA-A*23:01(0.15)	HLA-B*53:01 (0.4703)	HLA-A*24:02(0.01)	HLA-A*24:02(0.0180)	0.25258
		531	539	FAASVGFAR	HLA-A*68:01(0.15),	HLA-B*53:01 (0.2677)			0.03999
		313	321	RSHQFRAER	HLA-A*31:01(0.15)	HLA-B*57:01 (0.3629)			0.14818
		530	539	YFAASVGFAR	HLA-A*68:01(0.15), HLA-A*33:01(0.2)	HLA-B*53:01 (0.5216)			0.08473
		104	113	GQLNALLKAV	HLA-A*02:06(0.2)	HLA-B*57:01 (0.6311)			-0.15239
		423	431	GERFSFDSL	HLA-B*40:01(0.2)	HLA-B*53:01 (0.2919)	HLA-B*40:01(0.30)		0.00576
		497	505	FAYDVGVIR	HLA-A*68:01(0.2)	HLA-B*44:03 (0.1643)			0.20582
		109	117	LLKAVTDHY	HLA-B*15:01(0.2)	HLA-B*53:01 (0.4911)	HLA-B*15:01(0.30)		0.08373
		489	497	WLQEYGVAF	HLA-B*15:01(0.2)	HLA-B*44:03 (0.527)	HLA-B*15:01(0.40)		0.14915
		414	422	SYLQPFQLW	HLA-A*24:02(0.2)	HLA-B*44:03 (0.6599)	HLA-A*24:02(0.01)	HLA-A*24:02(0.0061)	-0.125
		273	281	QSLFYSLPW	HLA-B*58:01(0.2)	HLA-B*44:03 (0.468)	HLA-B*58:01(0.10)		-0.06097
		529	537	RYFAASVGF	HLA-A*23:01(0.25)	HLA-B*44:03 (0.1984)	HLA-A*24:02 (0.10)	HLA-A*24:02(0.0167)	0.01438
13	P35818, Type II secretion system protein D	596	605	MVFLRPTVVR	HLA-A*68:01(0.15)	HLA-B*35:01 (0.0667)			0.16194
		594	603	NLMVFLRPTV	HLA-A*02:01(0.2)	HLA-B*57:01 (.7367)			0.14978
		575	584	DIPLLGRLFR	HLA-A*33:01(0.2)	HLA-B*57:01 (0.9717),			0.1152
		277	285	RSANTRVIR	HLA-A*31:01(0.2)	HLA-B*57:01 (0.0888)			0.20531
		93	101	AQLSLSEVY	HLA-B*15:01(0.2)	HLA-B*57:01 (0.5207)	HLA-B*15:01(0.30)	HLA-B*15:01(0.0279)	-0.22798

		75	83	ETFVVDPRV	HLA-A*68:02(0.2)	HLA-B*44:03 (0.2039)			0.1615
		49	57	QEAHWTINL	HLA-B*40:01(0.25)	HLA-B*44:03 (0.105)	HLA-B*40:01(0.30)	HLA-B*40:01(0.1275)	0.40603
		50	58	EAHWTINLK	HLA-A*68:01(0.25)	HLA-B*53:01 (0.6433)			0.38453
		504	512	HINDGAALR	HLA-A*68:01(0.25)	HLA-B*53:01 (0.3047)			0.11659
		8	17	ALFAPSSRSY	HLA-B*15:01(0.25)	HLA-B*57:01 (0.9073)		HLA-A*03:01(0.1433)	-0.30324
14	Q9I3A9, Uncharacterized protein	487	495	YENDVDWRY	HLA-B*44:03(0.1)	HLA-B*53:01 (0.801)			0.29848
		694	702	LYFSAEVTF	HLA-A*23:01(0.15)	HLA-B*53:01 (0.8723)	HLA-A*24:02 (0.10)	HLA-A*24:02(0.0174)	0.0614
		113	122	SVYRPGLASV	HLA-A*02:03(0.15)	HLA-B*57:01 (0.711)		HLA-A*02:01(0.2802)	-0.001
		556	565	GLISDPLSVV	HLA-A*02:03(0.15)	HLA-B*57:01 (0.6296)		HLA-A*02:01(0.1215)	-0.23841
		398	406	RLLNGLSYR	HLA-A*31:01(0.15)	HLA-B*44:03 (0.146)	HLA-A*03:01(0.30)	HLA-A*03:01(0.0371)	-0.12933
		642	650	SLNGHRFER	HLA-A*31:01(0.15)	HLA-B*53:01 (0.196)		HLA-A*03:01(0.4492)	0.26952
		406	415	RYDQANSETF	HLA-A*24:02(0.15)	HLA-B*57:01 (0.5648)		HLA-A*24:02(0.0700)	-0.12611
		687	696	RYDSRHLLYF	HLA-A*24:02(0.15)	HLA-B*57:01 (0.8132)		HLA-A*24:02 (0.1162)	-0.09933
		271	279	NSDTLAHDY	HLA-A*01:01(0.15)	HLA-B*53:01 (0.8508)	HLA-A*01:01(0.01)		0.11255
		654	663	RLAKRIPLGK	HLA-A*03:01(0.15)	HLA-B*57:01 (0.9696)		HLA-B*08:01(0.2295)	-0.0243
		256	265	YAYRSPFPQV	HLA-A*02:06(0.2)	H-2-Kb(0.6443)			-0.08786
		48	57	AEVPGSISVL	HLA-B*40:01(0.2)	HLA-B*57:01 (0.7872)		HLA-B*40:01(0.0155)	-0.11608
		530	538	REIGYNGFF	HLA-B*40:01(0.2)	HLA-B*44:03 (0.4882)	HLA-B*40:01(0.10)	HLA-B*40:01(0.1365)	0.16461
		16	25	LLFGSKLAAA	HLA-A*02:03(0.2)	HLA-A*02:01 (0.5931)			-0.25356
		133	141	DVERIEVFR	HLA-A*68:01(0.2)	HLA-B*35:01 (0.1908)			0.4167
		531	539	EIGYNGFFR	HLA-A*68:01(0.2)	HLA-B*44:03 (0.1239)			0.20008
		466	474	LFNPQHSLR	HLA-A*31:01(0.2)	HLA-B*53:01 (0.5312)			-0.24171
		1	9	MPIASRAPV	HLA-B*07:02(0.2)		HLA-B*07:02(0.05)	HLA-B*07:02(0.0711)	-0.00327
		254	262	HPYAYRSPF	HLA-B*07:02(0.2), HLA-B*35:01(0.2)	HLA-B*44:03 (0.0733)	HLA-B*07:02(0.01)	HLA-B*07:02(0.0274)	-0.06281
		458	466	SPRLAVNYL	HLA-B*07:02(0.2)	HLA-B*07:02 (0.5578)	HLA-B*07:02(0.05)	HLA-B*07:02(0.0087)	0.07498
		311	319	NACEAGIAF	HLA-B*35:01(0.2)	HLA-B*44:03 (0.8723)			0.28843
		138	146	EVFRGPNTV	HLA-A*68:02(0.2)	HLA-B*53:01 (0.2236)		HLA-A*26:01(0.4431)	0.12986
		619	627	GSAGWMHD W	HLA-B*57:01(0.2)	HLA-B*53:01 (0.8763)	HLA-B*58:01(0.15)	HLA-B*58:01(0.0296)	0.13746
		496	504	RVRHLTPAP	HLA-A*30:01(0.2)	HLA-B*53:01 (0.5147)			0.08859
		406	415	RYDQANSETF	HLA-A*23:01(0.25)	HLA-B*57:01 (0.5648)		HLA-A*24:02(0.0700)	-0.12611
		687	696	RYDSRHLLYF	HLA-A*23:01(0.25)	HLA-B*57:01 (0.8132)		HLA-A*24:02(0.1162)	-0.09933
		539	547	RETGLAMDV	HLA-B*40:01(0.25)	HLA-B*44:03(0.3579)	HLA-B*40:01(0.30)	HLA-B*40:01 (0.2595)	-0.06251
		693	702	LLYFSAEVTF	HLA-B*15:01(0.25)	HLA-B*57:01 (0.5246)		HLA-A*24:02(0.1773)	0.14537
15	Q9I202, Pyroglutatmate porin OpdO	294	303	YPYIAGSDPY	HLA-B*35:01(0.1)	HLA-B*57:01 (0.3763)			0.06033
		319	327	RSWQLRYDY	HLA-A*30:02(0.15)	HLA-B*44:03 (0.598)	HLA-A*01:01(0.80)	HLA-B*58:01(0.2830)	0.0031
		255	263	SEDGGFREL	HLA-B*40:01(0.2)	HLA-B*53:01 (0.8133)	HLA-B*39:01(0.30)	HLA-B*39:01 (0.2621)	0.28668
		36	44	ELRNFYFNR	HLA-A*33:01(0.2)	HLA-B*44:03			0.21583

						(0.2008)			
		39	47	NFYFNRDFR	HLA-A*33:01(0.2)	HLA-B*44:03 (0.0497)			0.24614
		184	192	DYQVFSANR	HLA-A*33:01(0.2)	HLA-B*53:01 (0.2041)			-0.00855
		129	137	LLKVGALHF	HLA-B*15:01(0.2)	HLA-B*53:01 (0.7471)	HLA-B*15:01(0.05)	HLA-B*15:01(0.0899)	0.05091
		4	12	TPRLAAALL	HLA-B*07:02(0.2)	HLA-B*07:02 (0.4789)	HLA-B*07:02(0.20)	HLA-B*07:02(0.0177)	0.10711
		150	158	LPELFRGAL	HLA-B*07:02(0.2)	HLA-B*44:03 (0.0961)	HLA-B*07:02 (0.15)	HLA-B*07:02 (0.0367)	0.23552
		296	305	YIAGSDPYLV	HLA-A*02:06(0.25)	HLA-B*57:01 (0.5659)		HLA-A*02:01(0.3461)	-0.11274
		57	65	EWAQGFLLR	HLA-A*33:01(0.25)	HLA-B*44:03 (0.4547)			0.0235
		391	399	FANDLDENR	HLA-A*68:01(0.25)	HLA-B*53:01 (0.8757)			0.11102
		266	275	RAFGALFSLR	HLA-A*31:01(0.25)	HLA-B*57:01 (0.7654)			0.0635

Supplementary Table ST-4: MHC I epitopes shortlisting on the basis on antigenicity score and toxicity analysis

S.No	Protein ID, name	Start	End	Epitope	Antigenicity	Toxicity
1	Q9I6G3, Uncharacterized protein	193	201	VSNPALGAY	0.8041	Non-toxin
		467	475	LQAAGGPVL	0.6741	Non-toxin
		66	74	QTSRQDFTW	0.7864	Non-toxin
		542	550	LEASLGWRL	2.0409	Non-toxin
2	Q9HVJ4, Probable outer membrane protein	134	142	RTADEAGRY	1.0052	Non-toxin
		119	127	GEYGTRFSL	1.1993	Non- toxin
3	Q9I0E2, OpdB proline porin	173	181	IQAGRFTAF	-0.6029	Non-toxin
		137	146	GEMTVETPVF	1.2555	Non- toxin
4	Q9HYK0, Probable outer membrane protein	356	364	LAKVRAYEM	0.9886	Non- toxin
		199	207	RAAAARRTL	0.3949	Non- toxin
		222	230	APIERFPAL	0.1003	Non- toxin
5	Q9HYQ6, Patatin-like protein, PlpD	470	478	RYFVAPFLF	2.1857	Non- toxin
		476	484	FLFHEAQNV	-0.7472	Non- toxin
		181	189	LPQAIRASM	0.6620	Non- toxin
		476	484	FLFHEAQNV	-0.7472	Non- toxin
6	Q9I0W0, Outer membrane protein CzcC	334	342	LLRLRSEAV	1.2646	Non- toxin
		294	302	GERVNLIGL	-0.0870	Non- toxin
7	Q9I2I2, Probable TonB-dependent receptor	14	23	RLARAVPFLY	0.2510	Non- toxin
		522	530	LELGGGVDL	2.0270	Non- toxin
		68	77	RPVRDLQEAL	-0.6198	Non- toxin
		304	312	RTYRNRLER	0.8934	Non- toxin
		162	170	ITRRATDTW	0.5994	Non- toxin
		404	412	WESSPRLYL	0.9843	Non- toxin
		595	603	YALPAYSLW	0.9154	Non- toxin
		49	57	KLRDAPASV	0.6993	Non- toxin
		288	296	WSLAHNGQW	-0.8861	Non- toxin
8	Q9I4U9, Probable porin	325	333	RSWQLRYDY	2.9388	Non- toxin
		64	72	YESGYTEGL	1.5126	Non- toxin
		273	281	ALNALFTYR	0.6050	Non- toxin
		134	142	MPRLPVVQF	0.9824	Non- toxin
		308	316	PYLVNFVQI	-0.0193	Non- toxin
9	Q9I319, Type III secretion outer membrane protein PscC	120	128	RALTAAGIW	-0.3898	Non- toxin
		200	209	IEAPGIASIL	0.1449	Non- toxin
10	Q9I473, Probable tonB-dependent receptor	521	529	LPRRARRMF	-0.2420	Non- toxin
		93	101	TESDHVLVL	0.2701	Non- toxin
		401	409	TVSYGTAFK	0.0149	Non- toxin
		590	598	FGADHETAY	1.3888	Non- toxin
11	Q9I792, Uncharacterized protein	554	562	IYFRVDAFF	0.8002	Non- toxin
		529	537	RYFAASVGF	0.0201	Non- toxin
12	P35818, Type II secretion system protein D	49	57	QEAHWTINL	1.3769	Non- toxin
13	Q9I3A9, Uncharacterized protein	694	702	LYFSAEVTF	0.0854	Non- toxin
		530	538	REIGYNGFF	-0.0372	Non- toxin
		458	466	SPRLAVNYL	1.2239	Non- toxin
		619	627	GSAGWMHDW	-0.4238	Non- toxin
14	Q9I202, Pyroglutatmate porin OpdO	319	327	RSWQLRYDY	2.9388	Non- toxin
		255	263	SEDGGFREL	0.8272	Non- toxin
		129	137	LLKVGALHF	1.0811	Non- toxin
		4	12	TPRLAAALL	0.7871	Non- toxin
		150	158	LPELFRGAL	-0.6168	Non- toxin

Supplementary Table ST 5: identification of MHC II epitopes using IEDB server and epitopes toxicity and antigenicity analysis

S.No	Name	Start	End	MHC II epitopes	IEDB(percentile rank)	Toxicity	Antigencity
1	P35818, Type II secretion system protein D	240	254	GAQVIADARTNRLII	HLA-DRB1*03:01(0.09)	Non- toxin	0.2029
		328	342	ADESTNALVLLADPD	HLA-DQA1*01:02/DQB1*06:02(0.13)	Non- toxin	0.4299
		74	88	GETFVVDPRVKGQVS	HLA-DRB1*11:01(0.19)	Non- toxin	0.7776
2	Q9HU51, Uncharacterized protein	138	152	LTYYDYINKARLEQL	HLA-DRB1*11:01(0.16)	Non- toxin	0.0311
		116	130	SGREEYLKLLLNQEH	HLA-DRB1*04:05(0.26)	Non- toxin	0.2650
3	Q9I3A9, Uncharacterized protein	104	118	RLQVLIDGRSVYRPG	HLA-DRB1*03:01(0.03)	Non- toxin	0.4651
		665	679	ALELAGVLQQRLDDQ	HLA-DRB4*01:01(0.13)	Non- toxin	0.8109
		13	27	LLALLFGSKLAAADL	HLA-DQA1*01:02/DQB1*06:02(0.14)	Non- toxin	0.4423
4	Q9I6G3, Uncharacterized protein	300	314	MRDFSRLLTRLVLEQ	HLA-DRB1*11:01(0.04)	Non- toxin	-0.7649
		471	485	GGPVLLDQRLSGDTS	HLA-DRB1*03:01(0.05)	Non- toxin	0.7245
		33	47	LERLITDYPGRYRGT	HLA-DRB1*03:01(0.07)	Non- toxin	0.0651
5	Q9I202, Pyroglutatmate porin OpdO	266	280	RAFGALFSLRLGAHA	HLA-DRB1*09:01(0.01)	Non- toxin	1.0951
		182	196	SSDYQVFSANRIGGR	HLA-DRB1*07:01(0.24)	Non- toxin	0.6490
6	Q9I4U9, Probable porin	390	404	RNGTFRSDFGNDIDE	HLA-DRB3*01:01(0.04)	Non- toxin	0.0461
		58	72	QGFLLRYESGYTEGL	HLA-DRB1*15:01(0.14)	Non- toxin	0.7220
7	Q9I2I2, Probable TonB-dependent receptor	431	445	KAPSLKQLSPEYAAV	HLA-DRB1*09:01(0.03)	Non- toxin	0.6907
		10	24	LPPLRLARAVPFLYL	HLA-DRB3*02:02(0.06)	Non- toxin	1.0610
		536	550	WELNYTYLDARNRTA	HLA-DRB5*01:01(0.15)	Non- toxin	0.9702
8	Q9I319, Type III secretion outer membrane protein PscC	6	20	IGGLLALLPGAVLRA	HLA-DRB1*09:01(0.03)	Non- toxin	0.3371
		246	260	NAVVVRDHKDRLPMY	HLA-DRB1*03:01(0.07)	Non- toxin	-0.0549
		73	87	PQAFLQLMASLYNLG	HLA-DRB1*01:01(0.14)	Non- toxin	0.0204
9	Q9I792, Uncharacterized protein	519	533	GNAIELDARGRYFAA	HLA-DRB1*03:01(0.02)	Non- toxin	0.6998
10	Q9I0W0, Outer membrane protein CzcC						
11	Q9HVJ4, Probable outer membrane protein	204	218	DAKYRAGAAALSDRL	HLA-DRB1*09:01(0.02)	Non- toxin	0.7788
		453	467	TSRLRLAASLGRLGF	HLA-DRB3*02:02(0.08)	Non- toxin	0.1735
		427	441	MIELLNALTAYASAE	HLA-DQA1*01:02/DQB1*06:02(0.14)	Non- toxin	0.1514
		163	177	ATLQNTFALAAQAYY	HLA-DQA1*01:02/DQB1*06:02(0.16)	Non- toxin	0.5764
		453	467	TSRLRLAASLGRLGF	HLA-DRB5*01:01(0.18)	Non- toxin	0.1735
12	Q9HYK0, Probable outer membrane protein	405	419	AEARYAYLNAWLRLR	HLA-DRB5*01:01(0.01)	Non- toxin	0.7184
		359	373	VRAYEMAVAAAREQV	HLA-DRB5*01:01(0.05)	Non- toxin	0.3949
		145	159	REQVVLAEAQRRALE	HLA-DRB5*01:01(0.12)	Non- toxin	-0.0875
		84	98	ERDYRSYASTLSLEQ	HLA-DRB1*04:01(0.17)	Non- toxin	0.5761
13	Q9I0E2, OpdB proline porin						
14	Q9HYQ6, Patatin-like protein, PlpD	467	481	VGSRYFVAPFLFHEA	HLA-DPA1*01:03/DPB1*02:01(0.01)	Non- toxin	0.5079
		2	16	RRLLLVLLLLLPLSA	HLA-DPA1*03:01/DPB1*04:02(0.01)	Non- toxin	1.6032
		294	308	IDAGYRATTVLAARL	HLA-DQA1*01:02/DQB1*06:02(0.02)	Non- toxin	0.6612
		687	701	SGYINAASLMIGFDT	HLA-DQA1*01:02/DQB1*06:02(0.03)	Non- toxin	0.2508
		193	207	AVFAPVEIDGRLLVD	HLA-DRB1*03:01(0.07)	Non- toxin	0.3899
		5	19	LLVLLLLLPLSALAA	HLA-DRB1*01:01(0.14)	Non- toxin	1.3696
		586	600	RQWDLRLNKALSFGA	HLA-DRB3*02:02(0.16)	Non- toxin	1.3838
15	Q9I473, Probable tonB-dependent receptor	369	383	QLSLRRDDNQQFGVH	HLA-DRB3*01:01(0.01)	Non- toxin	0.8807
		232	246	DNGLELDGTLLRAKS	HLA-DRB1*03:01(0.03)	Non- toxin	1.0111
		212	226	EPDRDGYRNLSGNLR	HLA-DRB5*01:01(0.14)	Non- toxin	0.7545

Supplementary Table ST-6: Characterization of vaccine constructs using different server

S.No	Vaccine	AlgPred	ANTIGENpro	VaxiJen	SOLpro
1	VT1	Non-allergen	0.617893	1.5596	0.988354
2	VT2	Non-allergen	0.570354	1.5730	0.989859
3	VT3	Non-allergen	0.606306	1.7541	0.982037
4	VT4	Non-allergen	0.569399	1.5662	0.987401

Supplementary Table ST-7: Identification of docking score of HLA alleles and TLR4 complex with all vaccine constructs

S.No.	HLA alleles	VT1	VT3	VT4
1	HLA-DRB1*03:01 (1A6A)	-4.15	-11.56	-17.92
2	HLA-DRB1*15:01 (1BX2)	-18.84	-11.25	-22.69
3	HLA-DRB3*02:02 (3C5J)	-14.29	-6.73	-14.16
4	HLA-DRB5*01:01 (1H1S)	-32.90	-16.04	-5.26
5	HLA-B*44:03 (1SYS)	-10.93	-5.06	-32.07
6	HLA-B*53:01 (1A1M)	-1.44	-4.35	-20.03
7	HLA-B*15:01 (1XR8)	-17.53	-7.89	-11.61
8	HLA-B*39:01 (4O2E)	3.14	-1.97	-10.10
9	HLA-B*58:01 (5IM7)	-10.85	-31.91	-0.37
10	HLA-B*35:01 (1ZSD)	-2.54	-1.53	-10.10
11	TLR4/MD2 complex(2Z65)	-7.55	4.56	-6.52
12	TLR4/MD2 complex(3FXI)	0.63	2.92	-11

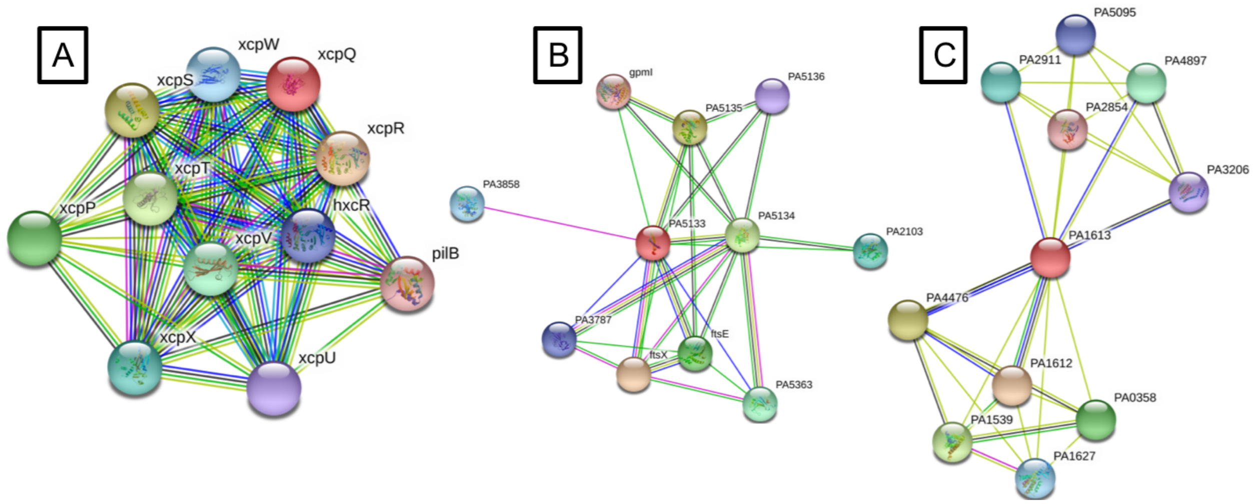

Supplementary Figure 1 Protein-protein interaction analysis of antigenic proteins using STRING server.

A) Protein ID P35818 intra species interacting partners are: xcpR General secretion pathway protein E, xcpS General secretion pathway protein F, xcpT General secretion pathway protein G, xcpP Secretion protein XcpP, xcpV General secretion pathway protein I, xcpX General secretion pathway protein K, xcpW General secretion pathway protein J, hxcR Type II secretion system protein, xcpU General secretion pathway outer membrane protein H, and pilB Type 4 fimbrial biogenesis protein PilB.

B) Protein ID Q9HU51, interacting partners are: ftsX Cell division protein FtsX, PA5135 Hypothetical protein, PA5134 Carboxyl-terminal protease, ftsE Cell division protein FtsE, PA5363 Hypothetical protein, PA2103 Molybdopterin biosynthesis protein MoeB, PA3858 Amino acid-binding protein, PA3787 Hypothetical protein, PA5136 Hypothetical protein, and gpmI Phosphoglyceromutase.

C) Protein ID Q9I3A9, partners are PA1612 Hypothetical protein, PA4476 Hypothetical protein, PA1539 Hypothetical protein, PA0358 Hypothetical protein, PA4897 Hypothetical protein, PA2911 TonB-dependent receptor, PA1627 Transcriptional regulator, PA5095 ABC transporter permease, PA3206 Two-component sensor, and PA2854 Hypothetical protein.

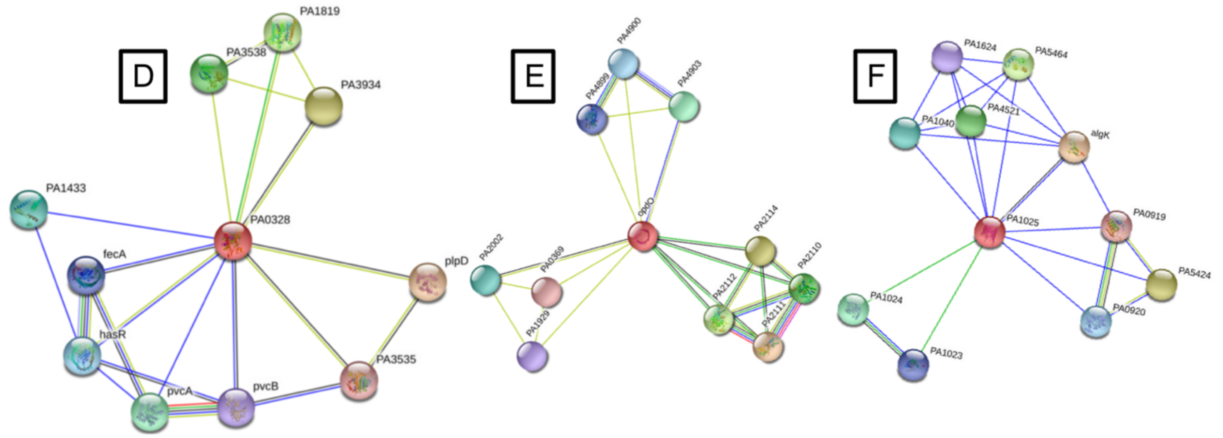

Supplementary Figure 1 Protein-protein interaction analysis of antigenic proteins using STRING server.

D) Protein ID Q9I6G3, intra-species interacting partners are plpD Patatin-like protein, PlpD, PA3934 Hypothetical protein, PA1819 Amino acid permease, PA3538 ABC transporter ATP-binding protein, pvcA Paerucumarin biosynthesis protein PvcA, PA1433 Hypothetical protein, hasR Heme uptake outer membrane receptor HasR, fecA Fe(III) dicitrate transporter FecA, pvcB Paerucumarin biosynthesis protein PvcB, and PA3535 Serine protease.

E) Protein ID Q9I202, interacting partners are PA2111 Hypothetical protein, PA2114 Major facilitator superfamily (MFS) transporter, PA2112 LamB/YcsF family protein, PA2110 Hypothetical protein PA4903 Major facilitator superfamily (MFS) transporter, PA2002 Hypothetical protein, PA4900 Major facilitator superfamily (MFS) transporter, PA4899 Aldehyde dehydrogenase, PA1929 Hypothetical protein and PA0369 Hypothetical protein.

F) Protein ID Q9I4U9, interacting partners are PA5424 Hypothetical protein, PA5464 Hypothetical protein, PA4521 Hypothetical protein, PA1024 2-nitropropane dioxygenase, PA1040 Hypothetical protein, PA0920 Hypothetical protein, PA1023 Short-chain dehydrogenase, PA1624 Hypothetical protein, and PA0919 Hypothetical protein

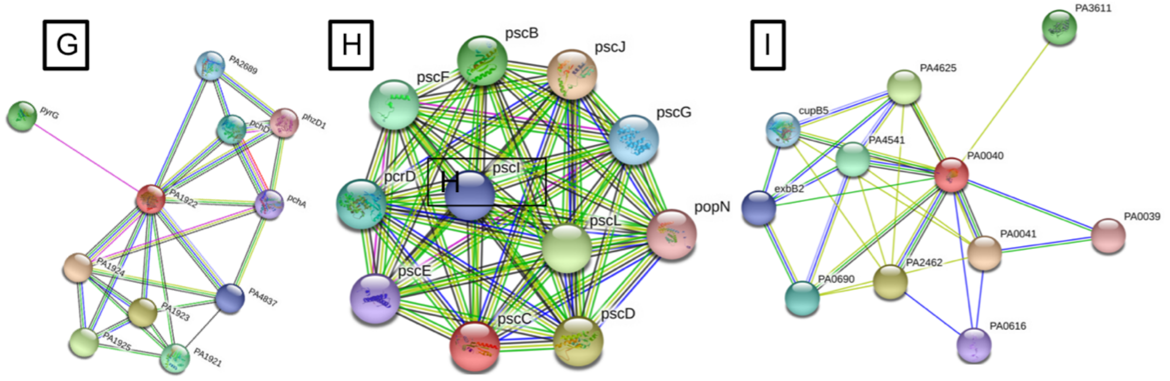

Supplementary Figure 1 Protein-protein interaction analysis of antigenic proteins using STRING server.

G) Protein ID Q9I2I2, interacting partners are PA1924 Hypothetical protein, PA1923 Cobaltochelatase subunit CobN, PA1925 Hypothetical protein, pyrG CTP, PA1921 Hypothetical protein, pchD Pyochelin biosynthesis protein PchD, PA2689 Hypothetical protein, PA4837 Hypothetical protein, pchA Salicylate biosynthesis isochorismate synthase and phzD1 Phenazine biosynthesis protein PhzD.

H) Protein ID Q9I319, interacting partners are pscJ Type III export protein PscJ, pscD Type III export protein PscD, pscL Type III secretion system protein, pscB Type III export apparatus protein, pscF Type III export protein PscF, pcrD Type III secretory apparatus protein PcrD, pscG Type III export protein PscG, pscI, Type III export protein PscI, pscE Type III export protein PscE, and popN Type III secretion outer membrane protein PopN.

I) Protein ID Q9I792 interacting partners PA0041 Hemagglutinin, PA2462 Hypothetical protein, PA4625 Hypothetical protein, PA3611 Hypothetical protein, PA4541 Hypothetical protein, PA0690 Hypothetical protein , cupB5 Adhesive protein CupB5, exbB2 Transporter ExbB2, PA0616 Hypothetical protein, and PA0039 Hypothetical protein.

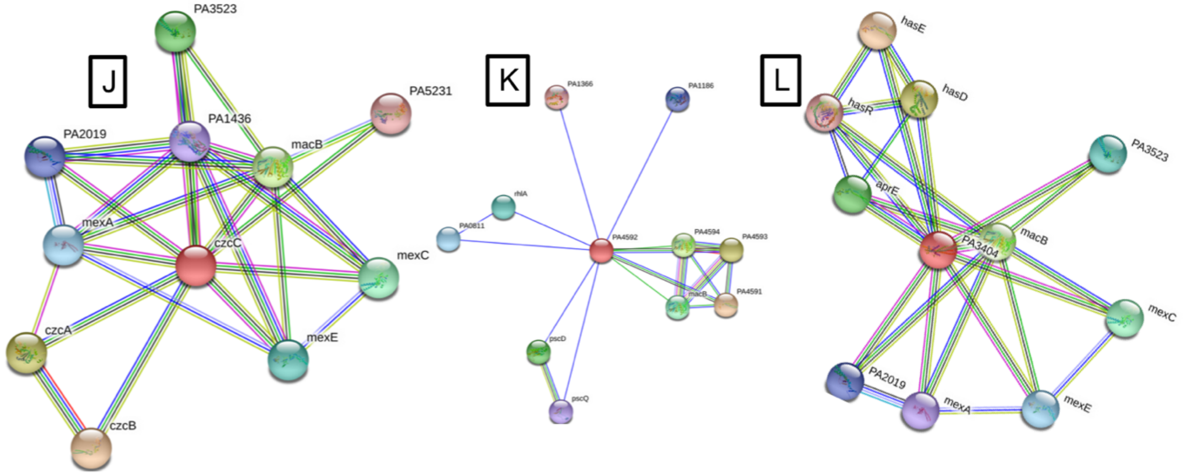

Supplementary Figure 1 Protein-protein interaction analysis of antigenic proteins using STRING server. **J)** Protein ID Q9I0W0 interacting partners are CzcB Resistance-nodulation-cell division (RND) divalent metal cation efflux membrane fusion protein CzcB, CzcA Resistance-Nodulation-cell Division (RND) divalent metal cation efflux transporter CzcA, MacB Protein PvdT, PA3523 Resistance-Nodulation-cell Division (RND) efflux membrane fusion protein, MexC Resistance-nodulation-cell division (RND) multidrug efflux membrane fusion protein MexC , MexE Resistance-nodulation-cell division (RND) multidrug efflux membrane fusion protein, MexE (mexA Resistance-nodulation-cell division (RND) multidrug efflux membrane fusion protein, MexA, PA2019 Periplasmic multidrug efflux lipoprotein, PA1436 Resistance-Nodulation-cell Division (RND) efflux transporter, and PA5231 ABC transporter ATP-binding protein/permease.

K) protein ID Q9HVJ4, interacting partners are PA4591 Hypothetical protein, PA4593 ABC transporter permease, PA4594 ABC transporter ATP-binding protein, pscD Type III export protein PscD, macB Protein PvdT, rhlA Rhamnosyltransferase chain A; Required for rhamnolipid surfactant production, PA0811 Major facilitator superfamily (MFS) transporter, PA1186 Hypothetical protein, pscQ Type III secretion system protein, and PA1366 Hypothetical protein. **L)** protein ID Q9HYK0 interacting partners are hasE Metalloprotease secretion protein, hasD Transporter HasD, macB Protein PvdT, aprE Alkaline protease secretion protein AprE, mexC Resistance-nodulation-cell division (RND) multidrug efflux membrane fusion protein MexC, PA3523 Resistance-Nodulation-cell Division (RND) efflux membrane fusion protein, mexE Resistance-nodulation-cell division (RND) multidrug efflux membrane fusion protein MexE, PA2019 Periplasmic multidrug efflux lipoprotein, mexA Resistance-nodulation-cell division (RND) multidrug efflux membrane fusion protein MexA, hasR Heme uptake outer membrane receptor HasR.

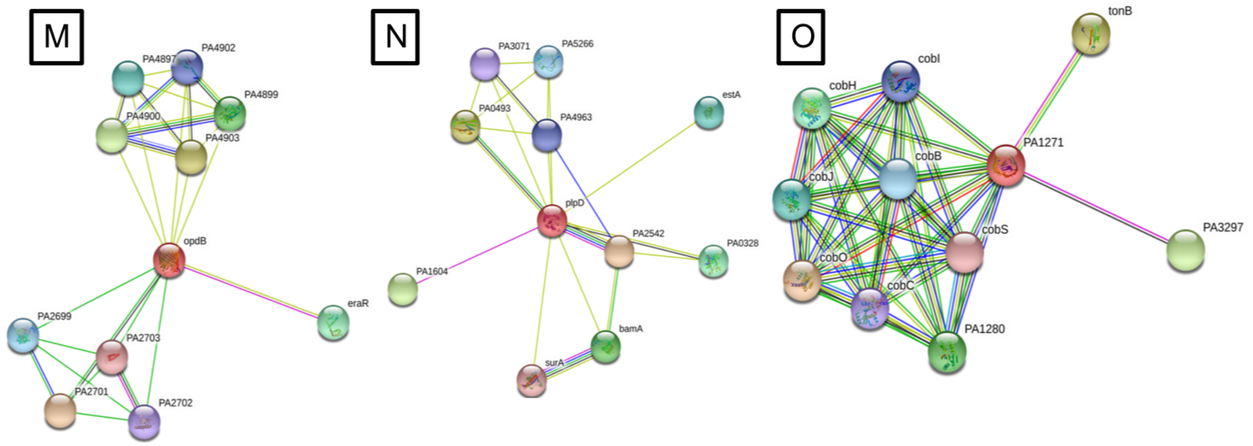

Supplementary Figure 1 Protein-protein interaction analysis of antigenic proteins using STRING server.

M) Protein ID Q9I0E2, interacting partners are PA2701 Major facilitator superfamily (MFS) transporter, PA4903 Major facilitator superfamily (MFS) transporter, PA4900 Major facilitator superfamily (MFS) transporter, PA4899 Aldehyde dehydrogenase , eraR Response regulator EraR, PA4897 Hypothetical protein, PA2699 Hypothetical protein, PA4902 Transcriptional regulator, PA2702 Hypothetical protein, and PA2703 Hypothetical protein

N) Protein ID Q9HYQ6 interacting partners are PA2542 Hypothetical protein PA0493 Hypothetical protein, PA1604 Hypothetical protein, bamA Outer membrane protein Opr86, PA0328 Hypothetical protein, estA Esterase, PA5266 Hypothetical protein, PA4963 Hypothetical protein, PA3071 Hypothetical protein, and surA Peptidyl-prolyl cis-trans isomerase SurA

O) Protein ID Q9I473 interacting partners are cobO cob(I)yrinic acid a,c-diamide adenosyltransferase, tonB Protein TonB1, PA3297 ATP-dependent helicase, PA1280 Hypothetical protein, cobH precorrin-8X methylmutase, cobJ Precorrin-3 methylase CobJ, cobB Cobyrinic acid a,c-diamide synthase, cobI Precorrin-2 C(20)-methyltransferase, cobC Threonine-phosphate decarboxylase, and cobS Cobalamin synthase.

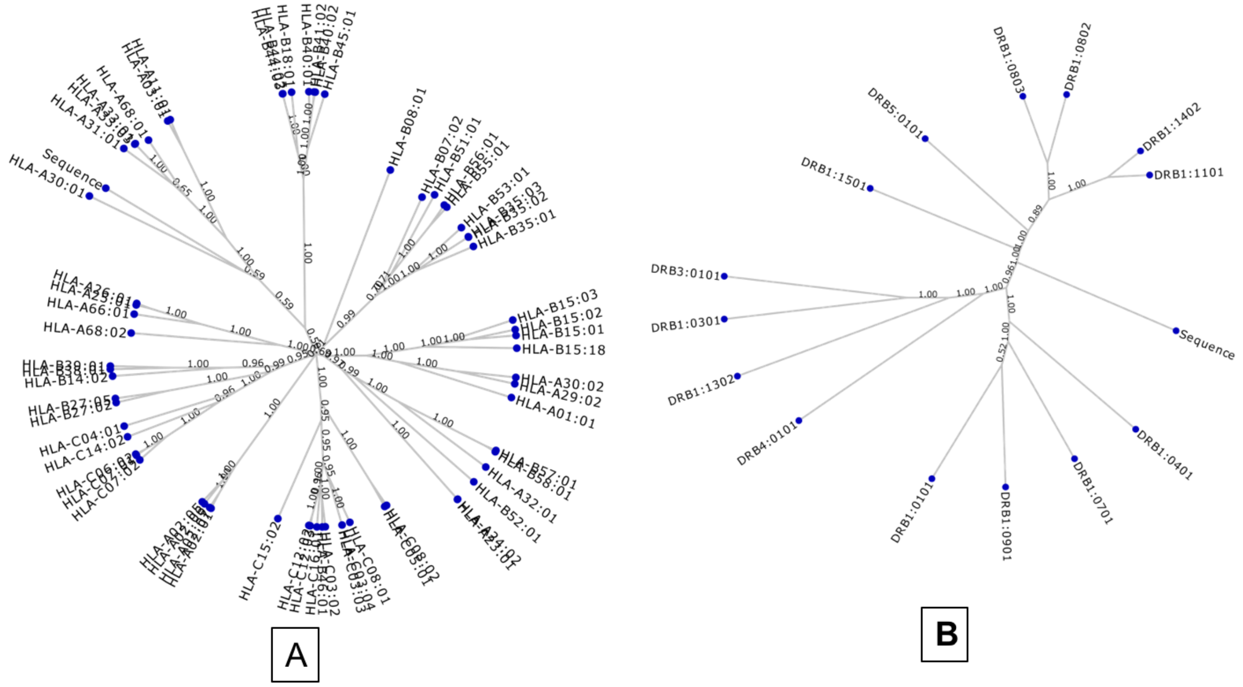

Supplementary Figure SF 2: Cluster analysis of the HLA alleles for both MHC molecules through tree map representation. **(A)** Representing the cluster of the MHC-I. **(B)** Representing the cluster of MHC-II molecules.
